# In Vitro and Preclinical Systematic Dose-Effect Studies of Auger Electron- and *β* Particle-Emitting Radionuclides and External Beam Radiation for Cancer Treatment

**DOI:** 10.1016/j.ijrobp.2024.05.017

**Published:** 2024-05-24

**Authors:** Ines M. Costa, George Firth, Jana Kim, Arshiya Banu, Truc T. Pham, Kavitha Sunassee, Sophie Langdon, Vittorio De Santis, Laurence Vass, Giuseppe Schettino, Gilbert O. Fruhwirth, Samantha Y.A. Terry

**Affiliations:** * Department of Imaging Chemistry and Biology, School of Biomedical Engineering and Imaging Sciences, King’s College London, London, United Kingdom; † Imaging Therapies and Cancer Group, Comprehensive Cancer Centre, School of Cancer and Pharmaceutical Sciences, King’s College London, London, United Kingdom; ‡ Medical Radiation Science Group, National Physical Laboratory, Teddington, United Kingdom

## Abstract

**Purpose:**

Despite a rise in clinical use of radiopharmaceutical therapies, the biological effects of radionuclides and their relationship with absorbed radiation dose are poorly understood. Here, we set out to define this relationship for Auger electron emitters [^99m^Tc]TcO_4_^–^ and [^123^I]I^–^ and *β*^–-^particle emitter [^188^Re]ReO_4_^–^. Studies were carried out using genetically modified cells that permitted direct radionuclide comparisons.

**Methods and Materials:**

Triple-negative MDA-MB-231 breast cancer cells expressing the human sodium iodide symporter (hNIS) and green fluorescent protein (GFP; MDA-MB-231.hNIS-GFP) were used. *In vitro* radiotoxicity of [^99m^Tc]TcO_4_^–^, [^123^I]I^–^, and [^188^Re]ReO_4_^–^ was determined using clonogenic assays. Radionuclide uptake, efflux, and subcellular location were used to calculate nuclear absorbed doses using the Medical Internal Radiation Dose (MIRD) formalism. *In vivo* studies were performed using female NSG mice bearing orthotopic MDA-MB-231.hNIS-GFP tumors and compared with X-ray−treated (12.6-15 Gy) and untreated cohorts. Absorbed dose per unit activity in tumors and sodium iodide symporter−expressing organs was extrapolated to reference human adult models using OLINDA/EXM.

**Results:**

[^99m^Tc]TcO_4_^–^ and [^123^I]I^–^ reduced the survival fraction only in hNIS-expressing cells, whereas [^188^Re]ReO_4_^–^ reduced survival fraction in hNIS-expressing and parental cells. [^123^I]I^–^ required 2.4- and 1.5-fold lower decays/cell to achieve 37% survival compared with [^99m^Tc]TcO_4_^–^ and [^188^Re]ReO_4_^–^, respectively, after 72 hours of incubation. Additionally, [^99m^Tc] TcO_4_^–^, [^123^I]I^–^, and [^188^Re]ReO_4_^–^ had superior cell killing effectiveness *in vitro* compared with X-rays. *In vivo*, X-ray led to a greater median survival compared with [^188^Re]ReO_4_^–^ and [^123^I]I^–^ (54 days vs 45 and 43 days, respectively). Unlike the X-ray cohort, no metastases were visualized in the radionuclide-treated cohorts. Extrapolated human absorbed doses of [^188^Re] ReO_4_^–^ to a 1 g tumor were 13.8- and 11.2-fold greater than for [^123^I]I^–^ in female and male models, respectively.

**Conclusions:**

This work reports reference dose-effect data using cell and tumor models for [^99m^Tc]TcO_4_^–^, [^123^I]I^–^, and [^188^Re]ReO_4_^–^ for the first time. We further demonstrate the tumor-controlling effects of [^123^I]I^–^ and [^188^Re]ReO_4_^–^ in comparison with external beam radiation therapy.

## Introduction

Therapeutic radiopharmaceuticals deliver radioactivity to the target of interest in both primary and disseminated disease.^1^ Some radionuclides investigated for molecular radiotherapy (MRT) can be considered theranostic because they emit *γ*-rays or positrons (*β*^+^), which can be exploited for single-photon emission computed tomography (SPECT) or positron emission tomography (PET) imaging, respectively.

The field of MRT has been driven by *β*^−-^particle-emitters, such as ([^177^Lu]Lu-prostate-specific membrane antigen-617 [^177^Lu]Lu-PSMA-617 for patients with advanced prostate cancer. Current MRT research builds on previous successes with radiopharmaceutical therapies including, [^131^I]I-Meta-Iodo-Benzyl-Guanidine ([^131^I]I-MIBG) for neuroblastoma in children, [^90^Y]Y-anti-CD20 antibodies for non-Hodgkin lymphoma, [^90^Y]Y-microspheres for hepatic tumors, and ^131^I for thyroid cancer.^2^ The success of MRT has largely been energized through its implementation in patients with advanced midgut neuroendocrine tumors and [Lu]Lu-[DOTA^0^-Tyr^3^]-octreotate ([^177^Lu]Lu-DOTA-TATE) is now a well-established therapeutic option.^3^ Brachytherapy using *β*^–^-emitting [^188^Re]Re-microspheres also has clinical potential in patients with hepatocellular carcinoma.^4^ Studies also suggest that ^188^Re could become a key isotope in targeted MRT, for example, as [^188^Re]Re-anti-CD20 for non-Hodgkin lymphoma^5^ or other targeted deemed useful for MRT.

The MRT radioisotope portfolio is currently expanding to include radionuclides that emit *α*-particles,^6–8^ beyond ^223^Ra, to Auger electron (AE)−emitting radionuclides.^9–13^ AE radiation therapy exploits the cytotoxicity of low-energy electrons emitted during radioactive decay that travel very short distances (typically <1 *μ*m, 4-26 keV/*μ*m).^14^ For example, ^99m^Tc(I) tricarbonyl complexes containing a triphenylphosphonium and bombesin peptide have proved effective at killing prostate cancer cells *in vitro* as did [^99m^Tc]Tc-labeled doxorubicin in HeLa, melanoma B16, and epidermoid carcinoma A-431 cancer cell lines.^15,16^ The potential of AE radiation therapy is also exemplified by interest in developing clinical dose escalation trials, for example, for [^111^In]In-diethylenetriamine pentaacetate-human epidermal growth factor ([^111^In]In-DTPA-hEGF) in patients with metastatic breast cancer,^17^ and advances toward treating patients with glioblastoma with an [^123^I]I-labeled poly (ADP-ribose) polymerase 1 (PARP1) inhibitor.^12^

Currently, there are few biological studies directly comparing the radiobiological effects of different radionuclides in the same model; most work in this area used bacterial plasmids.^18,19^ These assays do not account for any complex cellular environment or longer-distance effects, for example, crossfire effect. As such, few studies correlate radiobiological effects with the delivered radiation absorbed dose at the cellular level.^20,21^ This knowledge is key to reach a better understanding of the consequences of ionizing radiation exposure in biological matter, and ultimately boost radiopharmaceutical development for more effective MRT strategies for cancer treatment.

Here, we employed triple-negative breast cancer cells, previously engineered to express the human sodium iodide symporter (hNIS) in which [^99m^Tc]TcO_4_^-^ proved an effective radiotherapeutic.^20,22^ In this work, the same approach was used *in vitro* and *in vivo* to determine and compare the biological effect of 2 additional sodium iodide symporter substrates, [^123^I]I^–^ and [^188^Re]ReO_4_^–^, alongside X-ray radiation (external beam radiation therapy [EBRT]) as a comparator for which the biological effects relating to radiation absorbed dose are well understood.

## Methods and Materials

Reagents were purchased from Thermo Fisher Scientific, Corning, or Sarstedt, unless stated otherwise.

### Cell culture

MDA-MB-231.hNIS-GFP with hNIS-GFP long-term cell surface expression were generated previously.^22^ Parental MDA-MB-231 and MDA-MB-231.hNIS-GFP cells were cultured in Dulbecco’s Modified Eagle Medium (1 g/L glucose) supplemented with 10% fetal bovine serum (Invitrogen), 2 mM L-glutamine, 100 U/mL penicillin, and 100 *μ*g/mL streptomycin (Gibco; complete media) in a humidified 5% (v/v) CO_2_ atmosphere at 37°C. Cells were passaged using 0.5% trypsin containing 0.2% EDTA.

### Radionuclides and *in vitro* external beam irradiation

Na[^99m^Tc]TcO_4_ (^99m^Tc; half-life, 6.04 hours) in 0.9% sterile saline was supplied at 0.4 ± 0.1 MBq/*μ*L by the Guy’s and St Thomas’ NHS foundation Trust radiopharmacy. Na[^123^I]I ([^123^I]; half-life, 13.2 hours) was obtained at 0.05 ± 0.02 MBq/*μ*L (Curium). Na [^188^Re]ReO_4_ ([^188^Re]; half-life, 16.98 hours) was obtained at 0.08 ± 0.03 MBq/*μ*L in 0.9% saline by elution of a ^188^W/^188^Re generator (OncoBeta GmbH). To obtain daughter radionuclides [^99^Tc] TcO_4_^–^ (*β*^–^; half-life, 2.1 × 10^5^ years), ^123^Te (*β*^–^; half-life, 9.2 × 10^6^ years) and [^188^Os]OsO_4_^–^ (stable), 4 MBq/mL radionuclide solutions were decayed for 10 half-lives.

For *in vivo* studies, [^188^Re]ReO_4_^–^ was concentrated to 1.3 ± 0.1 MBq/*μ*L as described in the Appendix E1 (Fig. E1).^23^

*In vitro*, EBRT was delivered at 5 Gy/min using a gamma irradiator (Nordion International Inc). Dose delivered was validated using Gafchromic EBT3 films calibrated at the National Physical Laboratory.

### Cellular uptake and efflux

Cells seeded in 24-well plates (2 × 10^5^ cells/well, 1 mL complete cell medium) adhered overnight at 37°C in a humidified 5% (v/v) CO_2_ atmosphere. The medium was then refreshed and cells incubated with 0.2 MBq/mL [^99m^Tc]TcO_4_^–^, [^123^I] I^–^, or [^188^Re]ReO_4_^–^ (250 *μ*L) for up to 72 hours, or at increasing concentrations up to 4 MBq/mL for 30 minutes, at 37°C in a humidified 5% (v/v) CO_2_ atmosphere. Uptake specificity was demonstrated through blocking with hNIS substrate ClO_4_^−^ (12.5 *μ*M, 30 minutes). After incubation, the medium was collected, and cells were washed twice in phosphate-buffered saline (PBS) and lysed in 1 M NaOH. Radio-activity was measured using a gamma-counter (LKB-Wallac CompuGamma 1282) to determine intracellular radionuclide uptake (milli Bequerel per cell; mBq/cell).

For efflux studies, after incubation with [^99m^Tc]TcO_4_^–^, [^123^I]I^–^, or [^188^Re]ReO_4_^-^ (0.2 MBq/mL for 30 minutes), the medium was discarded and cells were washed and incubated in fresh medium for up to 5 hours. At indicated time points, the medium was collected and fresh medium added. At the last time point, the medium was collected, combined with PBS washes, and cells were lysed in 1 M NaOH. Radioactivity was gamma-counted and results expressed as percentage of original radionuclide uptake.

### Cytotoxicity and cellular dosimetry

Cells (2 × 10^5^ cell/well) in 24-well plates were incubated with increasing concentrations (0-4 MBq/mL) of [^99m^Tc] TcO_4_^–^, [^123^I]I^–^, or [^188^Re]ReO_4_^–^ (250 *μ*L) for 24 or 72 hours in a humidified 5% (v/v) CO_2_ atmosphere at 37°C. Decayed samples were used at highest concentrations only. Cells were also seeded at 0.5 × 10^5^ cells per petri dish and gamma-irradiated (up to 8 Gy).

After treatment, cells were washed with PBS. Harvested cells were then seeded into 6-well plates and cultured for 10 to 12 days. The medium was refreshed every 2 to 3 days. Plates were fixed and stained for 20 minutes with 1% crystal violet in methanol (Sigma-Aldrich), and colonies (≥50 cells) were counted. The survival fraction (SF) and cellular dosimetry were determined as per Appendix E1.

### Animal studies and tumor inoculation

Animal experiments were performed in accordance with the Animals (Scientific Procedures) Act of 1986, with protocols approved by UK Home Office (P9C94E8A4 and PP9982297) and local institute animal welfare and ethical review body under Home Office Project Licenses. MDA-MB-231.hNIS-GFP xenografts were established in 5- to 6-week-old female NOD.Cg-Prkdc^scid^Il2rg^tm1Wjl^/SzJ (NSG) mice (Charles River) by injecting 1 × 10^6^ cells into the mammary fat pad while mice were under anesthesia; cells maintained their hNIS expression throughout (Fig. E2). For more information regarding housing, tumor inoculation, monitoring, and humane endpoints, see Appendix E1.

### *In vivo* molecular and X-ray radiation therapy

Mice were randomized for *in vivo* therapy studies when hNIS-GFP-expressing MDA-MB-231 tumors reached 76 ± 21 mm^3^ (~day 19 post tumor inoculation). Treatment groups were (1) 55 MBq [^123^I]I^–^ or (2) 4.4 MBq [^188^Re]ReO_4_^–^ (n = 5-7 mice/group) in 120 *μ*L sterile saline or (3) EBRT (n = 6). Animals were under anesthesia (1.5%-2.0% isoflurane in O_2_; 1.0-1.5 L/min) during intravenous injection of the radionuclides or X-ray radiation therapy. For the radionuclide therapy groups, mice were imaged at various time points over 24 hours by SPECT/computed tomography (CT) (methods *vide infra*, n = 4/group). Remaining mice in groups 1 to 3 and untreated mice (group 4) received 120 *μ*L sterile saline and were imaged by CT only.

For *in vivo* X-ray radiation therapy studies, a small animal image-guided radiation therapy (SmART+) system with an integrated cone beam CT unit fitted with a 10 mm circular collimator and a 0.3 mm Cu filter was used (Precision X-Ray Inc). For further details, see Appendix E1 (example in Fig. E3).

### *In vivo* SPECT/CT imaging and image-based quantification and dosimetry

Radionuclides were delivered to mice as described above, and SPECT/CT scans were acquired over a period of 1.5 hours and at 5 hours and 24 hours after radiotracer administration. For more details, including on reconstruction and image-based quantification, see Appendix E1.

Time-activity curves (TACs) of the percentage of injected activity as a function of time post administration were determined (see Appendix E1) and inputted into the OLINDA/EXM software version 2.2.3 (Hermes Medical Solutions). This was then converted to absorbed dose per unit activity administered (milli Gray per mega Becquerel; mGy/MBq) as per Appendix E1.

### *Ex vivo* tissue analyses

Tissues and tumors were harvested from treated mice culled by cervical dislocation. hNIS expression was determined by fluorescence imaging of GFP signal using an IVIS Spectrum high-throughput imaging system (PerkinElmer) to detect GFP fluorescence (excitation, 500 nm; emission, 540 nm). *Ex vivo* immunohistochemistry and autoradiography were performed as per Appendix E1.

### Statistics

Normal distribution of data was verified using Shapiro-Wilk tests. Statistical significance was calculated with 1-way or 2-way analyses of variance with Tukey multiple comparisons test (5% significance level, GraphPad Prism v9.1.0) unless stated otherwise. Data is presented as average ± SD.

## Results

### *In vitro* uptake and efflux

**Fig. 1:**
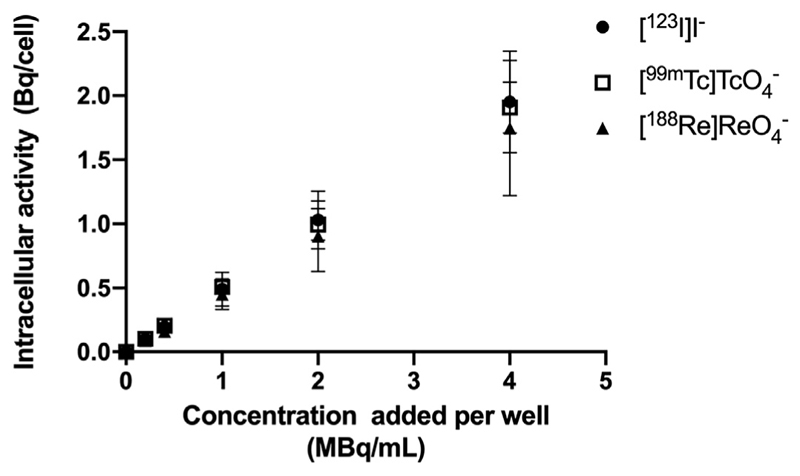
Intracellular radioactivity after 0.5-hour incubation of radionuclides in MDA-MB-231.hNIS-GFP cells (up to 4 MBq/mL). N = 3 to 4/group.

Cellular uptake of [^123^I]I^–^, [^99m^Tc]TcO_4_^–^, or [^188^Re]ReO_4_^–^ in MDA-MB-231.hNIS-GFP cells reached a plateau after 30 minutes (55.5% ± 7.5%, 46.5% ± 7.6%, and 50.3% ± 0.8% of administered activity, respectively), which was blocked when coincubated with NaClO_4_ (Fig. E4). Increasing radio-activity concentrations of [^123^I]I^–^, [^99m^Tc]TcO_4_^–^, and [^188^Re]ReO_4_^–^ resulted in a linear increase of cellular uptake (Fig. 1; Fig. E4B; *P* = .9943 between radionuclides). Here, maximum intracellular activity was 2000 ± 400, 1900 ± 200, and 1700 ± 400 mBq/cell for [^123^I]I^–^, [^99m^Tc]TcO_4_^–^, and [^188^Re]ReO_4_^–^, respectively, at 4 MBq/mL. Data showed first-order decay efflux kinetics for [^123^I]I^–^, [^99m^Tc]TcO_4_^–^, and [^188^Re]ReO_4_^–^ with an efflux half-life of 0.68 hours (95% CI, 0.57-0.83), 0.58 hours (95% CI, 0.47-0.72), and 0.50 hours (95% CI, 0.45-0.55), respectively (Fig. E5; *P* = .9333 between radionuclides).

### *In vitro* cell killing

**Fig. 2:**
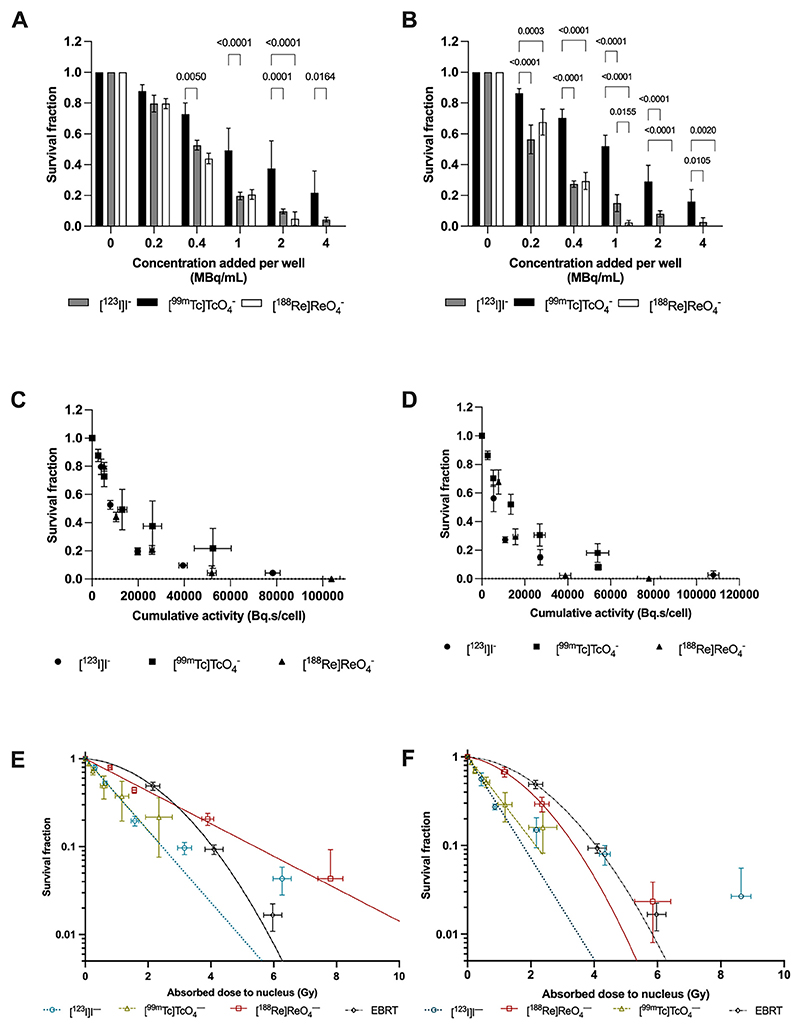
Survival fraction of MDA-MB-231.hNIS-GFP cells after (A, C) 24 hours and (B, D) 72 hours of treatment with [^123^I] I^–^, [^99m^Tc]TcO_4_^–^, and [^188^Re]ReO_4_^–^ plotted as a function of activity concentration per well (A, B) and cumulative activity (C, D). Dose-response curves of the survival fraction of MDA-MB-231.hNIS-GFP cells irradiated with external beam radiation therapy (EBRT) up to 8 Gy or incubated with ^[99m^Tc]TcO_4_^–^, [^123^I]I^–^, and [^188^Re]ReO_4_^–^ for (E) 24 hours and (F) 72 hours as a function of the estimated absorbed dose to nucleus (in Gray; Gy) using S_self_ and S_cross_ values. *β* was set at 0 for [^123^I]I^–^, [^99m^Tc] TcO_4_^–^, and [^188^Re]ReO_4_^–^ (at 24 hours only). N = 3 to 4/group.

After 24 hours of exposure, radioactivity concentrations of 0.62 MBq/mL [^123^I]I^–^ (89.5 mBq/cell; 95% CI, SF_0.32_ to SF_0.42_), 1.87 MBq/mL [^99m^Tc]TcO_4_^–^ (30.4 mBq/cell; 95% CI, SF_0.43_ to SF_0.31_), and 0.70 MBq/mL [^188^Re]ReO_4_^–^ (58.5 mBq/cell; 95% CI, SF_0.32_ to SF_0.42_) were calculated to reduce the SF of MDA-MB-231.hNIS-GFP cells to 37% (SF_0.37_ Fig. 2A). Furthermore, after incubation of MDA-MB-231. hNIS-GFP cells with [^123^I]I^–^ or [^188^Re]ReO_4_^–^ for 72 hours, SF was further reduced compared with 24 hours of incubation, respectively (both *P* < .0001); this was not observed for [^99m^Tc]TcO_4_^–^ (Fig. 2B). No toxicity was seen in parental MDA-MB-231 cells incubated with radionuclides, except for [^188^Re]ReO_4_^–^ or in MDA-MB-231.hNIS-GFP cells incubated with decayed radionuclide daughters (Figs. E6 and E7). Both at 24- and 72-hour incubation times, the number of decays per cell from [^123^I]I^–^ required to achieve SF_0.37_ was lower than for [^99m^Tc]TcO_4_^–^ and [^188^Re]ReO_4_^–^, respectively (Fig. 2C, D; Fig. E8; Table 1).

Dose-response curves, created using radiation absorbed dose measurements from S-values (Table E1) and subcellular localization data (Fig. E9), showed that the estimated delivered absorbed nuclear dose to reach SF_0.37_ was 1.06 ± 0.02 Gy, 1.15 ± 0.65Gy, and 2.34 ± 0.09 Gy for 24-hour incubation for [^123^I]I^–^, [^99m^Tc]TcO_4_^–^, and [^188^Re]ReO_4_^–^, respectively (Fig. 2E; Fig. E10). These values are lower than that for X-rays, which was 2.58 ± 0.16 Gy. Studies were carried out with EBRT as well as radionuclides to compare the latter to a treatment for which the dose-effect relationship is well-established. After 72 hours, the estimated delivered absorbed nuclear dose to reach SF_0.37_ for radionuclides was lower than that at 24 hours (Fig. 2F; Fig. E10). The *α*/*β* ratios in linear quadratic (LQ) models were 1.01 ± 0.27 Gy for EBRT and 11.07 ± 13.63 Gy for ^188^Re (at 72 hours of incubation only; Fig. 2; Fig. E10). For [^99m^Tc]TcO_4_^–^, [^123^I]I, and [^188^Re]ReO_4_^–^ (latter at only 24 hours of incubation), *β* was set to 0 to best fit data. The *α* value for [^99m^Tc]TcO_4_^–^ and [^188^Re]ReO_4_^–^ remained unchanged but that for [^123^I]I^–^ increased over time from 0.94 ± 0.01/Gy (24-hour incubation) to 1.33 ± 0.20/Gy. For [^188^Re]ReO_4_^–^, a *β* component became apparent at 72 hours of incubation only (Fig. 2; Fig. E10).

### *In vivo* dosimetry and therapeutic efficacy

[^123^I]I^–^, [^188^Re]ReO_4_^–^, or EBRT was administered/carried out to/on mice bearing MDA-MB-231.hNIS-GFP tumors, with tumor doses spanning 12 to 15 Gy for each radiation therapy type. Owing to its clinical potential and our *in vitro* results here, [^123^I]I was chosen over [^99m^Tc]TcO_4_^–^ as the lead AE-emitting radionuclide to be investigated *in vivo*.

**Fig. 3:**
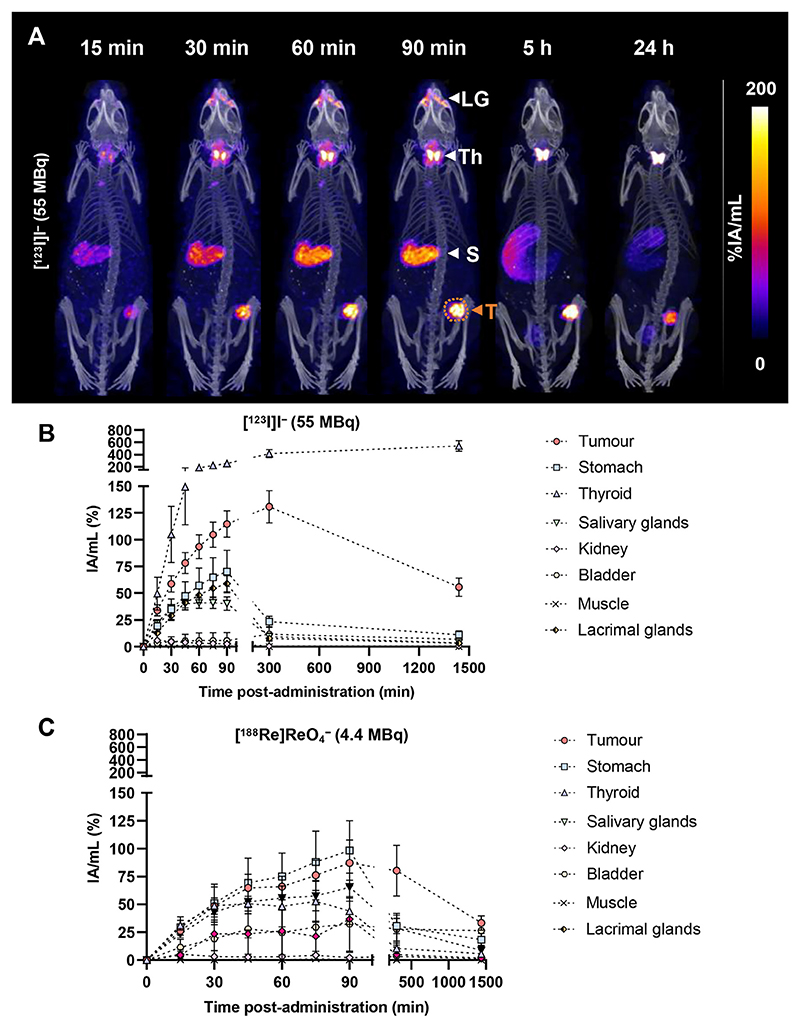
(A) Maximum intensity projections of single-photon emission computed tomography/computed tomography images of [^123^I]I^–^ in percentage activity per milliliter (%IA/mL) up to 24 hours after administration of 55 MBq [^123^I]I^–^. Image-based time-activity curves: %IA/mL up to 24 hours after administration of (B) [^123^I]I^–^ and (C) [^188^Re]ReO_4_^–^ in hNIS-GFP−expressing tumors and NIS-expressing organs. Time-activity curves were decay-corrected to time of radionuclide administration. N = 4 to 6 mice/group. *Abbreviations:* LG = lacrimal glands; S = stomach; T = MDA-MB-231.hNIS-GFP tumors; Th = thyroid.

**Fig. 4:**
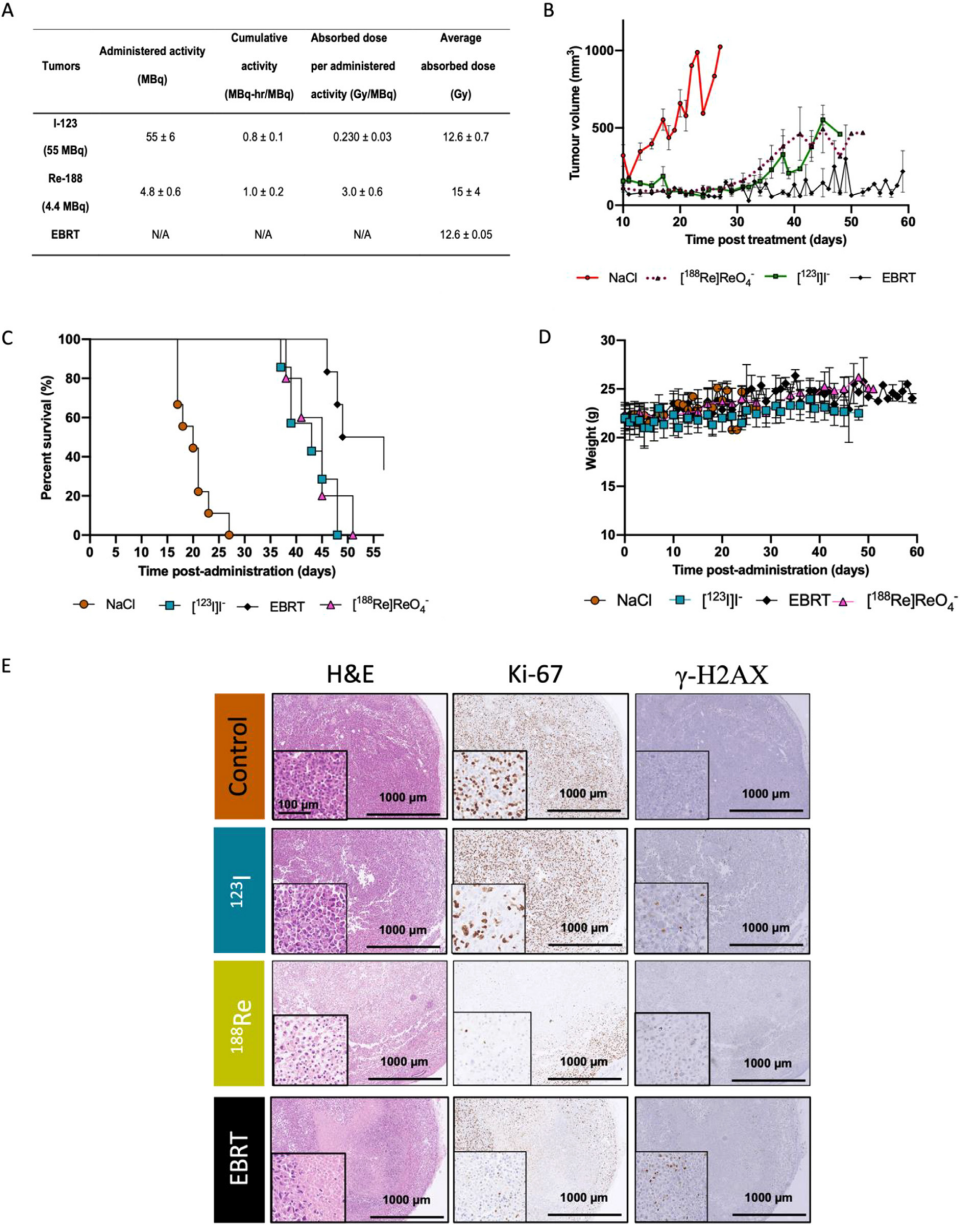
(A) Cumulative activity (MBq-hour/MBq) of [^123^I]I^–^ and [^188^Re]ReO_4_^–^, absorbed dose per unit activity administered to hNIS-GFP−expressing tumors (Gy/MBq), and respective absorbed dose (in Gray) (N = 4-6 mice/group), including for external beam radiation therapy (EBRT). Data are average ± SD. (B) Tumor volume growth (cubic millimeter) and (C) survival and (D) weight after intravenous administration of 55 MBq [^123^I]I^–^ (N = 7), 4.4 MBq of [^188^Re]ReO_4_^–^ (N = 5), and EBRT (N = 6) or administration of same volume of vehicle (NaCl) and/or computed tomography imaging only (N = 9) in an orthotopic MDA-MB-231. hNIS-GFP cancer model. (E) Immunohistochemistry confirms presence of radiotoxicity markers in tumor sections. Tumors were collected 8 days after administration of 55 MBq of [^123^I]I^–^, administration of 4.4 MBq of [^188^Re]ReO_4_^–^, or EBRT with X-ray. Sections were stained with hematoxylin and eosin (H&E), and for Ki-67 and *γ*H2AX. Scale bars are 100 *μ*m (in the box) and 1000 *μ*m. Gy/MBq = Gray per mega Becquerel. MBq-hour/MBq = mega Becquerel an hour per mega Becquerel. N/A = not applicable.

Both [^123^I]I^–^ and [^188^Re]ReO_4_^–^ were taken up by hNIS-GFP tumor xenografts and organs that express sodium iodide symporter, namely the thyroid, salivary glands, lacrimal glands, and stomach (Fig. 3A; Fig. E11). *Ex vivo* autoradiography and fluorescence imaging also showed that tumor cells took up the radionuclides and still expressed hNIS (Fig. E12). Time activity curves (TACs) were created (Fig. 3B, C) to quantify dose to the tumor (Fig. 4A) and healthy organs (Table E2). A single administration of [^123^I] I^–^ at 55 ± 6 MBq resulted in a tumor dose of 12.6 ± 0.7 Gy, whereas injection of [^188^Re]ReO_4_^–^ (4.8 ± 0.6 MBq) resulted in a tumor dose of 15 ± 4 Gy (Fig. 4A). EBRT was performed with a dose of 12.6 ± 0.05 Gy delivered to the tumor.

Tumor growth curves and Kaplan-Meier curves indicated a 2.2- to 2.3-fold improvement of median survival days after treatment with [^123^I]I^–^ or [^188^Re]ReO_4_^–^ (43 or 45 days, respectively) as opposed to 20 days observed for the control cohort (*P* < .0001; Fig. 4B, C); X-ray radiation therapy led to a median survival time of 54 (Fig. 4C). Therapies were well-tolerated (Fig. 4D). Tumor growth delay (Fig. 4B), decreased proliferation (Ki67), and enhanced DNA damage (*γ*H2AX; Fig. 4E) induced by X-rays were significantly greater those observed from radionuclide therapies (*P* < .0001).

Importantly, despite accurate delivery of X-ray radiation therapy to the primary tumors, with full-margin coverage (Fig. E3), GFP-positive metastases were visualized in all 6 mice; these were in the liver, bone, kidney, lungs, and axillary lymph nodes (Fig. E13). Metastases were also visible in 2 of 6 mice administered with [^188^Re]ReO_4_^–^, albeit in the liver only. Conversely, no metastases were overtly visible in the [^123^I]I^–^ cohort, although this was not directly looked for.

### Human dose extrapolation

Human absorbed doses per unit activity administered (milli Gray per mega Becquerel; mGy/MBq) were extrapolated from preclinical data and the International Commission on Radiological Protection 89 values (Table E3). Predicted tumor doses in humans were greater for [^188^Re]ReO_4_^–^ than for [^123^I]I^–^ and decreased with increasing tumor mass (eg, adult female model with [^123^I]I^–^, 1.1 ± 0.3 mGy/MBq vs 0.0019 ± 0.0005 mGy/MBq for a tumor with a mass of 0.1 and 100 g, respectively; Table 2). The greatest estimated absorbed dose per unit activity administered to healthy tissues delivered by [^188^Re]ReO_4_^–^ was to salivary glands (eg, 0.6 ± 0.2 mGy/MBq in male reference models), which was ~43-fold greater than that delivered by [^123^I]I^–^ (0.014 ± 0.003 mGy/MBq; Table E4).

Overall, the estimated absorbed doses were greater for [^188^Re]ReO_4_^–^ than for [^123^I]I^–^ for both adult female and male reference models. One exception was for [^123^I]I^–^, which delivered 0.7 ± 0.1 and 0.6 ± 0.1 mGy/MBq to the thyroid for adult female and adult male models, respectively, which was ~2-fold larger than that for [^188^Re]ReO_4_^–^. Notably, the main excretion route for both [^188^Re]ReO_4_^–^ and [^123^I]I^–^ is renal, and we found that estimated absorbed doses per unit activity administered were 0.09 ± 0.03 mGy/MBq for [^188^Re] ReO_4_^–^ and 0.0019 ± 0.0001 mGy/MBq for [^123^I]I^–^ in adult female models (Table E4). No statistical difference in absorbed radiation doses was observed when using female and male reference models.

## Discussion

Here, we showed that hNIS-expressing MDA-MB-231. hNIS-GFP cells could be used to comparatively investigate the biological effects of different theranostic radionuclides, that is, [^123^I]I^–^, [^99m^Tc]TcO_4_^–^, and [^188^Re]ReO_4_^–^. Relating absorbed radiation dose delivered to cells and tumors to cell killing and therapeutic efficacy, respectively, allowed us to infer the influence, if any, of differences in linear energy transfer. This work provides reference data and demonstrates the potential benefits of AE emitters. Importantly, we compare different radionuclides with different emissions with each other and X-ray radiation.

Only few studies have previously investigated comparative biological effectiveness of AEs, *β*^–^ or *α* emitters, and X-rays.^24–27^ For example, Boyd *et al*^28^ compared the induction of bystander effect by [^131^I]I-MIBG, [^123^I]I-MIBG, and [^211^At]At-MABG with that by EBRT. In addition, Runge *et al*^24^ used a rat thyroid cell line to compare how ^99m^Tc, ^188^Re, and ^223^Ra induced cell death. However, no dosimetry calculations within these were performed; hence, a dose-effect relationship comparing the different types of ionizing radiation remains elusive. Moreover, Freudenberg *et al*^29^ used rat thyroid cells to establish a relationship between cell survival and absorbed dose by AE emitters ^99m^Tc and ^123^I in comparison to X-ray irradiation. A limitation of that study was that cells metabolized iodide but not pertechnetate^30^; consequently, there was no balanced comparison between the 2 radionuclides.

In our work, we show, for the first time, that all 3 radio-nuclides had identical *in vitro* uptake and efflux and *in vivo* biodistribution kinetics in MDA-MB-231.hNIS-GFP tumor cells, which enabled interradionuclide comparisons as well as comparison with effects caused by external beam X-ray radiation. In this model, the uptake was hNIS-specific, and any biological effects observed could be attributed to radiation from administered radionuclides as their decay products did not cause toxicity. Moreover, the lack of toxicity from [^123^I]I^–^ and [^99m^Tc]TcO_4_^–^ on parental MDA-MB-231 cells highlighted the need for internalization of these radio-nuclides to be effective; this agrees with previous AE emitter data.^20^ This was not observed for the *β*^−^-emitter [^188^Re] ReO_4_^–^, although the *in vitro* therapeutic efficacy of [^188^Re] ReO_4_^–^ was greatest when accumulated into cells. Our data also demonstrated that, once internalized, nuclear targeting is not necessarily required for successful AE-mediated toxicity; this agrees with reports investigating ^125^I-based radiopharmaceuticals.^11,31,32^

Longer incubation, for example, 72 hours of incubation, revealed that there is an initial steep decrease in cellular SF when hNIS-GFP−expressing cells were incubated with [^123^I]I^–^ and [^99m^Tc]TcO_4_^–^. After incubation with [^123^I]I^–^, a subset of cells survived, even at nuclear absorbed doses between 4 and 8 Gy. This suggests that all cells need to be targeted to effectively eradicate all cancer cells and, consequently, that the available gammas or internal conversion electrons emitted by [^123^I]I^–^ were not causing a detectable crossfire effect. Unlike for the AE emitters, the dose-response curve for MDA-MB-231.hNIS-GFP cells incubated with [^188^Re]ReO_4_^–^ for 72 hours followed the LQ model as observed for MDA-MB-231.hNIS-GFP irradiated with EBRT with the “typical shoulder” and an *α*/*β* ratio of 2.07 ± 0.12 Gy.

Cellular dosimetry was carried out for spherical cells and not ellipsoid cells such as the MDA-MB-231 cells would have been during the *in vitro* assays. This is because MIRD-cell only allows dosimetry estimations based on spherical cells. Nonetheless, Monte Carlo studies have provided some information on the variation of the S-values for various geometric shapes/sizes and concluded that “….widely used approximation of spherical cells is reasonably accurate (within 20-30%) even for ellipsoidal geometries” (page 113).^33^ Use and development of a bespoke Monte Carlo code may have improved results but would have required extensive validation, which was outside the scope of the work reported here.

Here, we fitted the data using the LQ model, despite it perhaps not being appropriate for radionuclides because of the protracted radiation exposure, which is not accounted for in the LQ model and is interlinked with cell cycle distribution, DNA damage repair, and heterogeneous cell radio-sensitivity within a sample. Nonetheless, it currently remains the gold standard radiobiological model for cell survival. When mathematically fitting the LQ model onto the data from cells incubated with [^99m^Tc]TcO_4_^–^, [^123^I]I^–^, and [^188^Re]ReO_4_^–^ (at 24 hours only), the *β* value was calculated to be negative. This is an impossibility biologically, and so the *β* value was set to 0 where it then describes a scenario where, at low doses, there is negligible to no sublethal damage or repair thereof. Further work modeling low dose-effect relationships could benefit from delving into this more deeply.

Here, when determining a dose-effect relationship, we have focused our attention on the biological outcome of SF_0.37_. However other biological outcomes, for example, equivalent dose of 2 Gy X-ray fractions, relative biological effectiveness, and biological equivalent dose, do not necessarily follow the same trend. For example, when absorbed radiation dose is accumulated over 24 or 72 hours, the required dose to reach a given SF endpoint will depend on the endpoint itself, that is, whether it is high or low SF. Here, the dose required to achieve low SF is larger in 72 hours than in 24 hours, likely due to cell inactivation of potentially sensitive cells. At higher administered activities, beyond 24 hours, the dose loses its effectiveness to kill the “insensitive” or unaffected cells thus far. However, considering a relatively high SF endpoint, such as SF_0.37_ used here, the “extra” exposure time may help underpin the almost lethal dose already delivered during the first 24 hours to the “sensitive cells.” What is clear is that [^123^I]I^–^ and [^99m^Tc] TcO4^–^ showed superior therapeutic effectiveness *in vitro* compared with EBRT per nuclear absorbed dose.

From a therapeutic perspective, among the AE emitters, [^99m^Tc]TcO_4_^–^ proved the least viable option because its short half-life as well as fewer AEs emissions per decay (4.4 AEs/decay) limited long-term cancer cell control. Consequently, *in vivo* studies were performed here with [^123^I]I^–^ as the AE emitter of choice (13.7 AEs/decay) with a vision that in future, [^123^I]I^–^ would be attached to a tumor-targeting moiety as a true molecular radionuclide therapeutic. Notably, [^123^I]I^–^ is also the AE emitter of choice for a radiopharmaceutical currently progressing through the (pre)clinical pipeline as a potential future agent for glioblastoma treatment.^12^ In future, this approach might also prove effective when comparing AE and *β*^−^ emitters with *α*-emitters that can be taken up by hNIS, for example,^211^At, or other radionuclides using oxine as the delivery method, as has previously been done for ^111^In and ^67^Ga.^34^

For systematic studies, it is critical to ensure that the same absorbed radiation dose is delivered to tumors, instead of prioritizing administration of the same activity. For example, delivery of 55 MBq [^188^Re]ReO_4_^–^ resulted in toxicity and a rapid decline in animal weight (data not shown); animals were therefore culled. Here, *in vivo* absorbed dose was calculated through TACs and the OLINDA/EXM software; this is a common method that has tested and validated previously.^35^ Ultimately, administering [^123^I]I^–^ or [^188^Re]ReO_4_^–^ at activities to deliver the same average absorbed radiation dose to tumors resulted in a similar delay in tumor growth. In this way, the effect, or in our case, lack of effect from difference in linear energy transfer, can be observed. Importantly, there was a need for higher [^123^I]I^–^ activities to achieve the same average tumor absorbed dose and therapeutic effect as when using [^188^Re]ReO_4_^–^. This is a key limiting factor to consider when choosing this AE emitter as a therapeutic tool; absorbed dose to critical organs and photon/electron ratio emission^36^ determines the maximum administered activity to patients. EBRT proved more effective at control of primary tumor growth compared with systemically administered [^123^I]I^–^ and [^188^Re]ReO_4_^–^, but importantly, EBRT did not attenuate metastasis formation/outgrowth.

Extrapolating the preclinical absorbed radiation doses to tumors and healthy tissues to a clinical scenario should enable us to determine whether a theranostic approach might be close to toxicity limits for key tissues, such as the kidney or bone marrow. However, these limits are mostly still based on data derived from ^131^I. This work is thus an important step toward better understanding the radiation dose-biological effect relationship for other radionuclides, although the values presented here are only applicable to a scenario where [^123^I]I^–^ or [^188^Re]ReO_4_^–^ are injected as a standalone radionuclide. In addition, it becomes clear that it is worth exploring [^188^Re]ReO_4_^–^ (half-life, 16.9 hours) as a novel therapeutic in thyroid cancer by carrying out comparative studies with current therapeutic *β*-emitting [^131^I]I^–^ (half-life, 8 days), especially because the physical half-life might make the logistics around treating thyroid cancer more straightforward. This might also increase the quality of life for thyroid cancer patients by limiting measures needed when discharged from the hospital.

Based on the work presented here, it is likely that an approach using EBRT for effective bulk primary tumor control together with exploiting AE- or *β*^−^-emitting theranostic to prevent metastasis establishment/outgrowth would likely prove most effective for overall cancer response. A successful contribution of MRT to therapeutic outcome will most likely involve a personalized element to ensure optimal radiopharmaceutical uptake and toxicity in cancer cells only, and this personalization approach may require the use of imaging data to assess relevant MRT targets and the extent of their expression in each individual patient for the purpose of therapy planning.

## Conclusions

This work showcases the power of using hNIS models to permit systematic comparative radionuclide investigations, here exemplified with [^99m^Tc]TcO_4_^–^, [^123^I]I^–^, and [^188^Re] ReO_4_^–^. We also report reference data, for the first time, compared in cell and tumor models that were fully balanced for all radionuclides used. We further demonstrate the relative tumor-controlling effects of [^123^I]I^–^, [^188^Re]ReO_4_^–^, and EBRT, whereby we found MRT with [^188^Re]ReO_4_^–^ to be able to counteract tumor dissemination in this model.

## Supplementary Material

Supplementary Information

## Figures and Tables

**Table 1:** Concentration (mega Becquerel per mL; MBq/mL), intracellular activity (milli Becqueral per cell; mBq/cell), and cumulative activity (decays per cell) to reduce the survival fraction for MDA-MB-231.hNIs-GFP cells to 0.37 after incubation for 24 and 72 hours with [^123^I]I^–^, [^99m^Tc]TcO_4_^–^, and [^188^Re]ReO_4_^–^

72 hours of incubation								
Radionuclide	Concentration added per well			Intracellular activity			Cumulated activity	
	MBq/mL	95% CI		mBq/cell	95% CI		Decays/cell	95% CI
^123^I	0.70	0.32-0.42		58.5	0.32-0.42		13,821	0.32-0.42
^99m^Tc	1.87	0.31-0.43		30.9	0.31-0.43		24,421	0.31-0.43
^188^Re	0.62	0.32-0.42		89.5	0.32-0.42		16,107	0.32-0.42
**72 hours of incubation**								
**Radionuclide**	**Concentration added per well**			**Intracellular activity**			**Cumulated activity**	
	**MBq/mL**	**95% CI**		**mBq/cell**	**95% CI**		**Decays/cell**	**95% CI**
^123^I	0.35	0.32-0.42		2.1	0.33-0.41		9497	0.24-0.50
^99m^Tc	1.41	0.33-0.41		N/A	N/A		22,419	0.25-0.49
^188^Re	0.38	0.30-0.44		6.3	0.30-0.44		14,946	0.07-0.57

Data are values interpolated from least-square fitting of the average of 3 biological repeats and respective 95% CI. N/A = not applicable.

**Table 2:** Human-extrapolated absorbed dose per unit activity administered to tumors (mGy/MBq) for adult female and male adult reference models accordingly to International Commission on Radiological Protection 89

Tumor (g)	Human-extrapolated absorbed dose per unit activity administered (mGy/MBq)	
	Adult female	
	^123^I	^188^Re
0.1	1.1 ± 0.3	13 ± 4
1	0.13 ± 0.03	1.7 ± 0.5
10	0.015 ± 0.004	0.2 ± 0.1
100	0.0019 ± 0.0005	0.021 ± 0.006
	**Adult male**	
**Tumor (g)**	**^123^I**	**^188^Re**
0.1	0.8 ± 0.2	8 ± 2
1	0.1 ± 0.02	1.1 ± 0.3
10	0.03 ± 0.03	0.12 ± 0.03
100	0.0014 ± 0.0003	0.013 ± 0.003

## Data Availability

Data is available upon request from corresponding authors.
