# Supplementary material for: In Vitro and Preclinical Systematic Dose-Effect Studies of Auger Electron- and β Particle-Emitting Radionuclides and External Beam Radiation for Cancer Treatment
Source: Int J Radiat Oncol Biol Phys. Author manuscript; Available in PMC 2024 Nov 29. (PMC7616868; doi:10.1016/j.ijrobp.2024.05.017)
Supplement: Supplementary Information [file EMS200083-supplement-Supplementary_Information.docx]

**supplementary information**

**supplementary methods**

***Concentrating [^188^Re]ReO_4_^─^***

For *in* vivo studies, [^188^Re]ReO_4_^─^ was concentrated as described previously (1). Briefly, this method is based on a two column tandem flow-through system that enables the separation of chloride anions in the eluting sterile saline solution from [^188^Re]ReO_4_^─^ eluted from the generator. The eluate containing [^188^Re]ReO_4_^─^ is first passed through a silver cationic cartridge (PS-Ag+ Chromafix SPE cartridge) onto an anion exchange cartridge (Sep-pak Light QMA 46 mg Sorbent per Cartridge, 40 µm particle size, Waters, UK). [^188^Re]ReO_4_^─^ was eluted with saline in 100 µL fractions. Radioactivity in each fraction was measured using a dose calibrator (Capintec CRC® – 25R, Ramsey, NJ, USA). The fractions containing the most concentrated [^188^Re]ReO_4_^─^ (fractions 3-4, Figure S1) were retained for animal studies. This method provided access to ^188^Re concentrations of 1.3±0.1 MBq/µL.

***Subcellular distribution***

For cellular fractionation studies, 2.5 x 10^6^ MBA-MB-231.hNIS-GFP cells were incubated with 0.2 MBq/mL [^99m^Tc]TcO_4_^─^, [^123^I]I^─^ or [^188^Re]ReO_4_^─^ in suspension (250 µL final volume) for 0.5 hour. After incubation, the supernatant was collected, cells were washed with PBS twice, and cell pellets were fractionated using a kit as per manufacturer’s instructions (Cell Signaling Technology). Radioactivity in the cytoplasmic, membrane/organelles and nuclear fractions was quantified using a gamma-counter. Radioactivity in the cytoplasmic and membrane/organelles fractions was combined and named hereafter as extra-nuclear compartment. Data were expressed as percentage activity in the extra-nuclear compartment and nuclear compartment.

***Cellular survival***

The survival fraction (SF) was calculated by accounting for the plating efficiency at each treatment and normalized to untreated groups (set at 1.0). For radionuclide-treated cells, clonogenicity was shown as a function of radioactivity concentration added per well (MBq/mL) and decays per cell up to 24- and 72-hours incubation.

***Cellular dosimetry***

Time activity curves were fitted on data from uptake and efflux assays that accounted for the exponential growth of cells and physical decay of the radionuclides over time using GraphPad Prism v9.1.0. The area under the curve was estimated using trapezoidal integration and corresponds to the cumulated activity (Bq-s) per cell, hereafter referred as decays per cell. Using MIRDcell V2.0 (2), S_self_-values for cytoplasm and nucleus source volumes and S_cross_-values were used to estimate the absorbed dose in the nuclear target volume per unit activity inside each source volume (Gy/Bq-s), e.g. nucleus or cytoplasm (Table S1).

S-values were obtained assuming concentric spherical cells and nuclei with a radius of 11 ± 2 and 6 ± 1 μm, respectively. These values were calculated from GFP (attached to hNIS, on the cell membrane) and Hoechst-33342 (incorporated into the DNA) confocal fluorescence imaging of live cells (A1R Nikon Eclipse Ti-E Inverted confocal microscope). The dimensions calculated here are in line with findings in the literature also (12.4  ±  2.1 µm in diameter; (3)). Following the MIRD formalism, [^99m^Tc]TcO_4_^─^, [^123^I]I^─^ and [^188^Re]ReO_4_^─^ were assumed to accumulate uniformly across all cells and within the cellular source compartments.

For the cross-dose, S_cross_-values as a function of distance between cells (0 - 1.5 cm as max distance in the multiwell plate used) were estimated using MIRDcell V2.0 (Table S1). The average number of cells at any given distance was then estimated based on the total number of cells plated and assuming a uniform random distribution in the multiwell plate. Finally, the measured cumulative activity per cell was used according to the following equation:

D_cross_ = Σ_d_ ( Ã x S_cross_(d) x N_cells_(d) )

where D_cross_ (in Gy) is the absorbed dose from the cross-fire contribution, Ã (in Bq s / cell) is the average cumulative activity per cell over the 24 hr or 72 hr, S_cross_(d) (in Gy / Bq s) is the S-value from a neighbour.

The surviving fraction (SF) was fitted using GraphPad Prism v9.1.0 to the linear-quadratic cell death model (LQ-model) with the constrain β=0 for [^99m^Tc]TcO4^─^, [^123^I]I^─^ and [^188^Re]ReO_4_^─^ data and β>0 for EBRT data and [^188^Re]ReO_4_^─^ data following 72 hours incubation.

***Animal studies***

Animal experiments were performed in accordance with the Animals (Scientific Procedures) Act. 1986, with protocols approved by UK Home Office and King’s College London (KCL) animal welfare and ethical review body (St Thomas’ Campus) under Home Office Project Licenses. Mice were female and 6-to-8-weeks old at experimental beginning.

Mice were maintained within the KCL Biological Services Unit under specific pathogen-free conditions in a dedicated and licensed air-conditioned animal room (at 23 ± 2°C and 40 - 60% relative humidity) under light/dark cycles lasting 12 hours/day. They were kept in individually ventilated standard plastic cages (IVC; 501 cm^2^ floor space; from Tecniplast) including environmental enrichment and bedding material in the form of sterilized wood chips, paper stripes and one cardboard roll per cage. Maximum cage occupancy was five animals, and animals were moved to fresh cages with fresh environmental enrichment and bedding material twice per week. Sterilized tap water and food were available ad libitum; food was PicoLab Rodent Diet 20 (LabDiet) in the form of 2.5 x 1.6 x 1.0 cm oval pellets that were supplied at the top of the cages.

Studies were terminated when mice reached endpoints of the therapeutic study. Humane endpoints were determined based on signs of animal discomfort or pain, significant weight loss (20% weight loss in the absence of other clinical signs or 15% weight loss in the presence of other clinical signs), when tumor volumetric size reached 500 mm^3^ or at 59 days post radionuclides injection or X-ray irradiation. Mice were culled via cervical dislocation while under anaesthesia.

***Tumor inoculation***

MDA-MB-231.hNIS-GFP cells were harvested, washed twice with PBS, and resuspended in PBS at 20 x 10^6^ cells/mL. An aliquot of cells was analysed for stable GFP fluorescence (and thus hNIS expression) before tumor inoculation (see Supplementary Methods and Figure S2). MDA-MB-231.hNIS-GFP xenografts were established in 5-6 weeks old female NOD.Cg-Prkdc^scid^Il2rg^tm1Wjl^/SzJ (NSG) mice (Charles River, UK) by injecting 1x10^6^ cells into the mammary fat pad, with mice under anaesthesia (1.5-2.0% isoflurane, O_2_ flow rate of 1.0-1.5 L/min). Mice were monitored and tumor volumes measured by calliper using V = ½ × (L× W^2^), with “L” being the longest diameter of the tumor and “W” the longest tumor diameter perpendicular to L.

***Flow cytometry of cells used for tumor inoculation***

An aliquot of cells (~1x10^6^ cells/mL) was analysed for GFP fluorescence before tumor inoculation. The remaining cells following tumor inoculation were also analysed to determine whether hNIS-GFP expression was affected during tumor establishment procedure. Cells were diluted with 2% (w/v) BSA and 2 mM EDTA solution and sieved through a 35 μm cell strainer snap cap into 5 mL round-bottom polystyrene tubes with a 35 μm cell strainer snap cap (FalconTM 352235, FisherScientific) to avoid doublets. Cells were vortexed before being loaded onto the BD FACSMelodyTM equipped with a 488 nm laser (emission: 527/32 BP). Parental MDA-MB-231 cells were used to determine the laser power and gating strategies. 20,000 single cell events were recorded per sample and data was analysed using FlowJo v.10.6.2.

***In vivo X-ray radiotherapy***

For in vivo X-ray radiotherapy studies, a small animal image-guided radiation therapy (SmART+) system with an integrated cone-beam CT unit fitted with a 10 mm circular collimator and a 0.3 mm Cu filter was used (Precision X-Ray Inc., Madison, CT). For further details, see Supplementary Methods. Mice were placed in a supine position and the temperature of the irradiator chamber was kept at 35^○^C using a heating lamp to keep mice warmed. Cone beam CT images of the tumors were acquired for each mouse using a 2 mm aluminium filter and the mouse soft tissue high dose CT pre-set (40 kVp, 8 mA, 1x1 binning, 0.1 mm voxels), which delivered an estimated dose to the centre-of-mass of the mouse of 12 cGy. The Monte Carlo irradiation treatment plan was created using SmART-ATP (Advanced Treatment Planning) Scientific Solutions software v2.0.20201216 (example in Figure S3). Tumors were irradiated with an absorbed dose of 12.6 ± 0.05 Gy across two beams (parallel-opposed beam pair from the anterior-posterior/posterior-anterior directions at 225 kVp, 20 mA). Dose delivery was performed across an average irradiation time of 96 ± 4 s for anterior/posterior beam (0 degrees) and 79.5 ± 0.9 s for posterior/anterior beam (180 degrees) according to dosimetry performed by the manufacturer during commissioning of the unit. Non-irradiated mice underwent CT imaging only. Mice were allowed to recover from anaesthesia and monitored (health and tumor growth) as described above.

***In vivo SPECT/CT imaging***

For imaging, anaesthetized mice were placed in prone position on the warmed (37^○^C) nanoSPECT/CT scanner bed (Mediso, Hungary), with anaesthesia maintained until the end of each imaging session. Radionuclides were delivered as described above. SPECT scans were acquired over a period of 1h 30 min (6 x 15 min sequential SPECT scans, 15 sec per frame, 360° rotation, low energy multi-pinhole with 1 mm aperture and 9 holes and 20% energy window centred on photo-peaks of ^123^I (159 keV: 143-175 keV) and ^188^Re (155 keV: 140-171 keV). At the end of the sequential SPECT scans, a single 20 min CT scan (55 kVp X-ray, exposure time 1000 ms, 360^○^ rotation and pitch 1 was performed. Mice were then allowed to completely recover from anaesthesia, and then re-anaesthetized and re-scanned by SPECT/CT at 5 hours and 24 hours after radiotracer administration. At 5 hours post administration, mice underwent a 45-minute SPECT scan (same acquisition parameters, but with 60 seconds per frame) followed by a 20-minute CT scan. At 24 hours post administration, mice underwent a 2-hour SPECT scan (same acquisition parameters, but 150 sec per frame) followed by a 20-minute CT scan.

All SPECT projections were reconstructed using Tera-Tomo^TM^ iterative algorithm (Mediso, Hungary; reconstruction (64 iterations, high regularization filter) with attenuation and scatter correction. Data was visualized and quantified using VivoQuant© v3.5-patch2 software (Invicro, Massachusetts, USA). Volumes of interest were manually delineated on the stomach, thyroid, salivary glands, lacrimal glands, hNIS-expressing tumors, kidney, bladder, and muscle. CT scans were used as an anatomic reference to define the boundary of the organs and tumors. SPECT/CT images were shown as maximum intensity projections (MIPs) and data was expressed as percentage of injected activity per mL (%IA/mL) using GraphPad Prism 9.1.0. All images were decay-corrected to the time of radiotracer administration.

After the imaging protocol detailed above, mice were longitudinally monitored, and tumor volume and body weight were measured.

***In vivo image-based dosimetry***

The activity in each NIS-expressing organ (thyroid glands, lacrimal glands, salivary glands, and stomach) and excretion organs (bladder and kidneys) for each individual mouse was normalized to the total administered activity to obtain the ‘percent injected radioactivity’ (%IA) taking into account the physical decay of ^123^I and ^188^Re. Time-activity-curves (TAC) of %IA as a function of time (h) post administration were plotted using Microsoft Excel and the area under the curve (AUC; cumulative activity) was calculated for each mouse and organ. The AUC was obtained by fitting a trapezoidal approximation up to the peak of the curve and then a mono-exponential fit thereafter. The obtained area under the curve, equal to the cumulative activity (MBq-hours per MBq administered), was inputted into the OLINDA/EXM® software version 2.2.3 (Hermes Medical Solutions). This was then converted to absorbed dose per unit activity administered (mGy/MBq), using a reference model of a 25 g mouse.

For tumors, salivary glands, and lacrimal glands, the absorbed dose per unit activity administered to these tissues was estimated using the sphere model. The mass of the organs and tumors (g) input into the OLINDA/EXM® software were obtained from the volumes of interest drawn on the acquired CT images (mm^3^) assuming a density of water (1 g/cm^3^). Absorbed dose (Gy) was calculated by multiplying the absorbed dose per unit activity administered (mGy/MBq) with administered activity (MBq).

***Ex vivo histology and autoradiography***

Four mice (N=1/group) were administered with either 55 MBq [^123^I]I^─^, 4.4 MBq [^188^Re]ReO_4_^─^, saline or treated with EBRT at 12.6 Gy. Mice underwent the same anaesthetic period and CT imaging as in the therapy studies. After 8 days post injection, mice were culled via cervical dislocation and hNIS-GFP tumors were collected, washed with PBS, and kept overnight in 4% (v/v) paraformaldehyde in PBS (#J19943-K2, Thermo Scientific^TM^) at 4^○^C. After three washes with PBS, tissues were transferred to tissue processing/embedding cassettes and stored at 70% ethanol (EtOH). Fixed tissues were processed and embedded in paraffin (at UCL IQPath (UCL Institute of Neurology, London, UK). Two sets of three adjacent tissue sections of 5 µm were sliced at approximately 800 µm between the two sets. The three adjacent sections were stained with haematoxylin & eosin (H+E) for morphologic evaluation of tissue pathology, Ki-67 to visualize tissue proliferation and γ-H2AX to visualize DNA damage. Sections were imaged with a Hamamatsu Nanozoomer S630 digital slide scanner.

Additional mice (N=2, 1 mouse/group) were administered with 55 MBq of [^123^I]I^─^ and 4.4 MBq of [^188^Re]ReO_4_^─^ and culled 1 hour post administration for autoradiography studies. Tumors and salivary glands were collected and embedded in O.C.T. (Optimal Cutting Temperature compound, #361603E, VWR). Frozen tissues were then sectioned at 10 µm thickness using a cryostat (MNT, SLEE medical). Different consecutive slices were exposed to phosphor imaging plates for up to 5 min and scanned using Amersham^TM^ Typhoon^TM^ biomolecular imaging (GE healthcare) with 25 µm pixel size.

***Ex vivo tissue hNIS analyses***

Mice were culled via cervical dislocation at times specified above and tissues were harvested. Tumor tissues were harvested and photographed. In addition, liver, lungs, stomach, salivary glands and kidneys were also collected from mice that had undergone EBRT or intravenous administration of 4.4 MBq of [^188^Re]ReO_4_^─^. Tumors and organs of interest were washed with PBS, dry-blotted on filter paper, and placed onto petri dishes. Thereafter, *ex vivo* fluorescence imaging of GFP signal was performed using an IVIS® Spectrum high-throughput imaging system (PerkinElmer®, USA) with appropriate settings to detect GFP fluorescence with an excitation of 500 nm and emission of 540 nm.

***Human dose extrapolation from mouse data***

To extrapolate the preclinical absorbed dose data to humans, the %IA of the radionuclide in organs of mice was converted to human %IA using the Kirschner equation (Equation 1):

$${\%IA}_{human}=\left[ \frac{\%IA \times m_{WB}}{m_{organ}} \right]_{animal} \times\left[ \frac{m_{organ}}{m_{WB}} \right]_{human}$$

**Equation 1** with %IA_human_ = percentage injected activity in a human, m_wb_ = mass of whole body, m_organ_ = mass of organ.

Animal %IA and organ masses were taken from imaging-based biodistribution data. Human organ masses and the whole-body weight were considered for reference adult female (60 kg) and reference adult male (73 kg) models according to International Commission on Radiological Protection (ICRP) publication 89 (4), which were input into the OLINDA/EXM^®^ software v2.2.3. Table 3 shows the weights of the human organs used for calculation of human equivalent injected activity via Equation 1.

The human extrapolated time-activity curves and respective area under curve (cumulative activity (MBq-hr per MBq administered)) were obtained as for mice (image-based dosimetry). Estimated cumulative activities were input into the OLINDA/EXM^®^ software to derive human organ absorbed dose per unit activity administered (Gy/MBq) for the NIS-expressing organs stomach, lacrimal glands, salivary glands and excretion source organs kidney and bladder. The mass of the tumor from each xenograft animal was extrapolated to a 1 g human tumor to obtain time-activity curves. Absorbed dose per unit activity administered (mGy/MBq) to human tumors was estimated using the sphere model assuming human tumors with masses of 0.1, 1, 10 and 100 g.

**supplementary tables**

**Table E1.** S-values for spherical cells with a cell and nucleus radii of 11 ± 2 μm and 6 ± 1 μm, respectively. S_self_- (top) and S_cross_- (bottom) values were taken from MIRDcell v.2. software.

| **Subcellular localisation**  **of radionuclides** | **Source compartment** | **Target volume** | **S_self_-values**  **(Gy/Bq-s)** | | |
| --- | --- | --- | --- | --- | --- |
|  |  |  | **I-123** | **Tc-99m** | **Re-188** |
| Nuclear compartment | Nucleus | Nucleus | 9.74x10^-4^ | 4.96x10^-4^ | 3.75x10^-4^ |
| Extra-nuclear compartment | Cytoplasm | Nucleus | 5.77x10^-5^ | 1.41x10^-5^ | 5.72x10^-5^ |

|  | **^188^Re** | **^123^I** | **^99m^Tc** |
| --- | --- | --- | --- |
| **Distance between cells** | **S_cross_-value** | **S_cross_-value** | **S_cross_-value** |
| **[μm]** | **[Gy/Bq s]** | **[Gy/Bq s]** | **[Gy/Bq s]** |
| 22 | 7.24E-06 | 1.80E-06 | 1.13E-06 |
| 44 | 1.77E-06 | 3.98E-07 | 2.89E-07 |
| 66 | 7.90E-07 | 1.85E-07 | 1.36E-07 |
| 88 | 4.76E-07 | 1.10E-07 | 8.19E-08 |
| 110 | 2.43E-07 | 7.57E-08 | 5.70E-08 |
| 132 | 1.69E-07 | 5.75E-08 | 4.47E-08 |
| 154 | 1.24E-07 | 4.75E-08 | 3.91E-08 |
| 176 | 9.48E-08 | 4.35E-08 | 4.20E-08 |
| 198 | 7.65E-08 | 4.89E-08 | 1.62E-08 |
| 220 | 6.48E-08 | 1.22E-08 | 3.30E-09 |
| 242 | 5.63E-08 | 2.91E-09 | 5.15E-09 |
| 264 | 4.50E-08 | 3.04E-09 | 1.53E-11 |
| 286 | 3.41E-08 | 4.62E-09 | 7.97E-13 |
| 308 | 2.59E-08 | 8.01E-10 | 7.67E-13 |
| 330 | 2.16E-08 | 2.33E-12 | 4.71E-13 |
| 352 | 1.94E-08 | 2.06E-12 | 4.64E-13 |
| 374 | 1.82E-08 | 1.83E-12 | 5.90E-13 |
| 396 | 1.48E-08 | 1.64E-12 | 2.59E-13 |
| 418 | 1.35E-08 | 1.47E-12 | 2.58E-13 |
| 440 | 1.27E-08 | 1.33E-12 | 3.01E-13 |
| 462 | 1.07E-08 | 1.21E-12 | 1.46E-13 |
| 484 | 9.84E-09 | 1.11E-12 | 1.45E-13 |
| 506 | 9.23E-09 | 1.02E-12 | 1.57E-13 |
| 660 | 5.24E-09 | 6.16E-13 | 5.07E-14 |
| 704 | 4.39E-09 | 5.47E-13 | 2.52E-14 |
| 880 | 2.72E-09 | 3.65E-13 | 6.93E-15 |
| 990 | 2.10E-09 | 2.98E-13 | 3.14E-15 |
| 1540 | 9.12E-10 | 1.75E-13 |  |
| 1980 | 4.62E-10 |  |  |
| 3080 | 1.53E-10 |  |  |
| 4400 | 5.62E-11 |  |  |
| 5500 | 2.34E-11 |  |  |
| 6600 | 8.72E-12 |  |  |
| 7777 | 3.91E-12 |  |  |

**Table E2.** Calculated average absorbed dose per unit activity administered (Gy/MBq) and total absorbed dose (Gy) delivered in this study to NIS-expressing organs and excretion organs.

| Organs | Average absorbed dose per unit activity administered  (Gy/MBq) | | Average absorbed dose  (Gy) | |
| --- | --- | --- | --- | --- |
|  | **I-123**  **(55 MBq)** | **Re-188**  **(4.4 MBq)** | **I-123**  **(55 MBq)** | **Re-188**  **(4.4 MBq)** |
| Thyroid | 1.0 ± 0.1 | 0.15 ± 0.05 | 55.0 ± 6.0 | 0.7 ± 0.3 |
| Salivary glands | 0.006 ± 0.005 | 0.6 ± 0.1 | 0.4 ± 0.3 | 1.0 ± 0.05 |
| Stomach | 0.06 ± 0.04 | 2.3 ± 1.3 | 3.2 ± 2.0 | 11.0 ± 5.0 |
| Lacrimal glands | 0.004 ± 0.002 | 0.11 ± 0.01 | 0.2 ±0.2 | 0.5 ± 0.1 |
| Bladder | 0.03 ± 0.02 | 0.5 ± 0.6 | 1.6 ± 1.2 | 5.1 ± 2.0 |
| Kidney | 0.01 ± 0.01 | 0.13 ± 0.02 | 0.6 ± 0.4 | 0.6 ± 0.2 |

**Table E3.** Weight (g) of the human organs of the reference adult female and male models. Values available on the OLINDA/EXM^®^ software v.2.2.3 were taken from the International Commission on Radiological Protection (ICRP) publication 89 (4).

| **Organ** | **ICRP 89 values** | |
| --- | --- | --- |
|  | **Adult Female (g)** | **Adult Male (g)** |
| **Thyroid** | 17 | 20 |
| **Salivary glands** | 70 | 85 |
| **Stomach** | 140 | 150 |
| **Lacrimal glands** | 15 | 15 |
| **Kidney** | 275 | 310 |
| **Urinary Bladder** | 40 | 50 |

**Table E4.** Human-extrapolated absorbed dose per unit activity administered (mGy/MBq) to NIS-expressing organs and excretion organs for adult female and male adult reference models accordingly to ICRP 89.

|  | Human-extrapolated absorbed dose per unit activity administered  (mGy/MBq*)* | |
| --- | --- | --- |
| Organs | **Adult Female** | |
|  | **Re-188** | **I-123** |
| Thyroid | 0.3 ± 0.1 | 0.7 ± 0.1 |
| Salivary Glands | 0.7 ± 0.3 | 0.017 ± 0.003 |
| Stomach Wall | 0.3 ± 0.1 | 0.014 ± 0.007 |
| Lacrimal Glands | 0.24 ± 0.08 | 0.017 ± 0.002 |
| Urinary Bladder Wall | 0.05 ± 0.03 | 0.002 ± 0.001 |
| Kidney | 0.09 ± 0.03 | 0.0019 ± 0.0001 |
| Organs | **Adult Male** | |
|  | **Re-188** | **I-123** |
| Thyroid | 0.28 ± 0.05 | 0.6 ± 0.1 |
| Salivary Glands | 0.6 ± 0.2 | 0.014 ± 0.003 |
| Stomach Wall | 0.3 ± 0.1 | 0.013 ± 0.002 |
| Lacrimal Glands | 0.22 ± 0.04 | 0.015 ± 0.003 |
| Urinary Bladder Wall | 0.04 ± 0.02 | 0.002 ± 0.001 |
| Kidney | 0.08 ± 0.02 | 0.0014 ± 0.0001 |

**supplementary figures**

**Figure E1.** **Percentage of activity of [^188^Re]ReO_4_^─^ collected in each fraction (100 µL/fraction) from the total activity trapped in the Sep-pak Light QMA carbonate cartridge.** 3^rd^ and 4^th^ fraction were kept for intravenous administration of [^188^Re]ReO_4_**^─^** to mice. Data is shown as average ± SD, (*N* = 4).


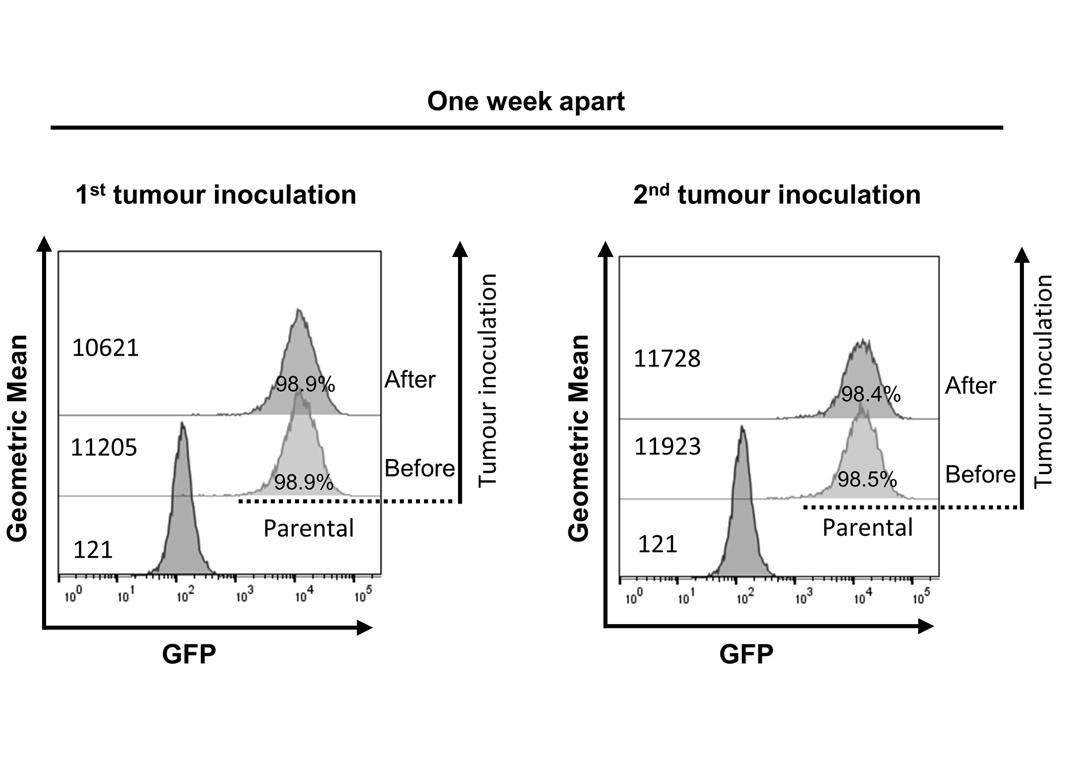


**Figure E2.** **Flow cytometry analysis showing stability of hNIS-GFP expression between tumor establishment procedures.** Stability of cells enabled tumor inoculations of MDA-MB-231.hNIS-GFP cells one week apart. hNIS-GFP expression was also not affected during the procedure of MDA-MB-231.hNIS-GFP xenograft establishment.

**
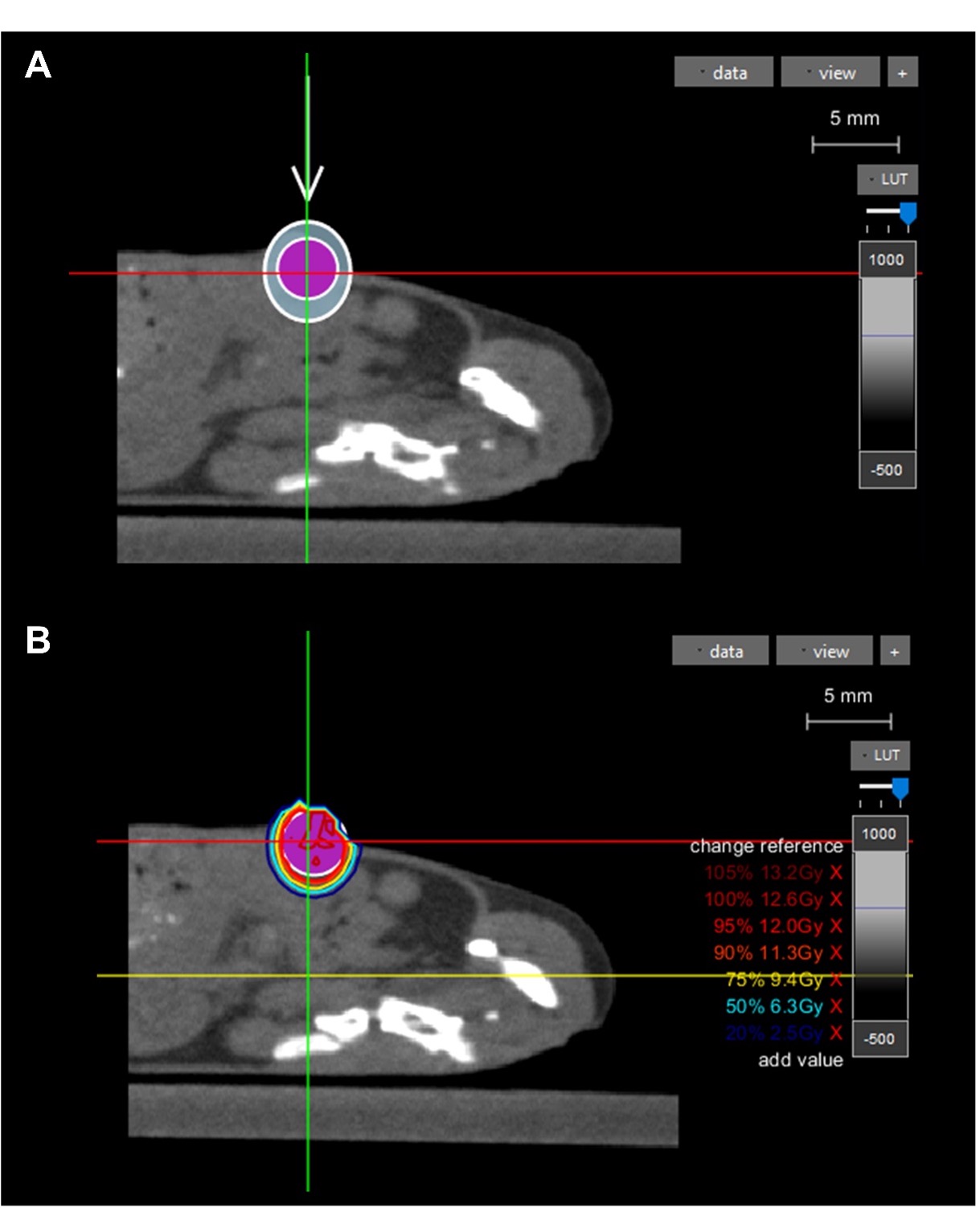
**

**Figure E3.** Representative screenshot of a treatment plan for irradiation of hNIS-expressing xenografts. For each (A) Direction of the irradiation beam in the sagittal view of the CT scan. Position of the beams were set up according to the position the tumors and (B) Monte Carlo algorithm was used to estimate the absorbed dose in the tumor and surrounding tissues. Screenshot of with the aim to deliver the desired dose to tumors, whilst minimising healthy tissue irradiation. Purple circle is the isocentre; grey circle is the radiation beam.

**
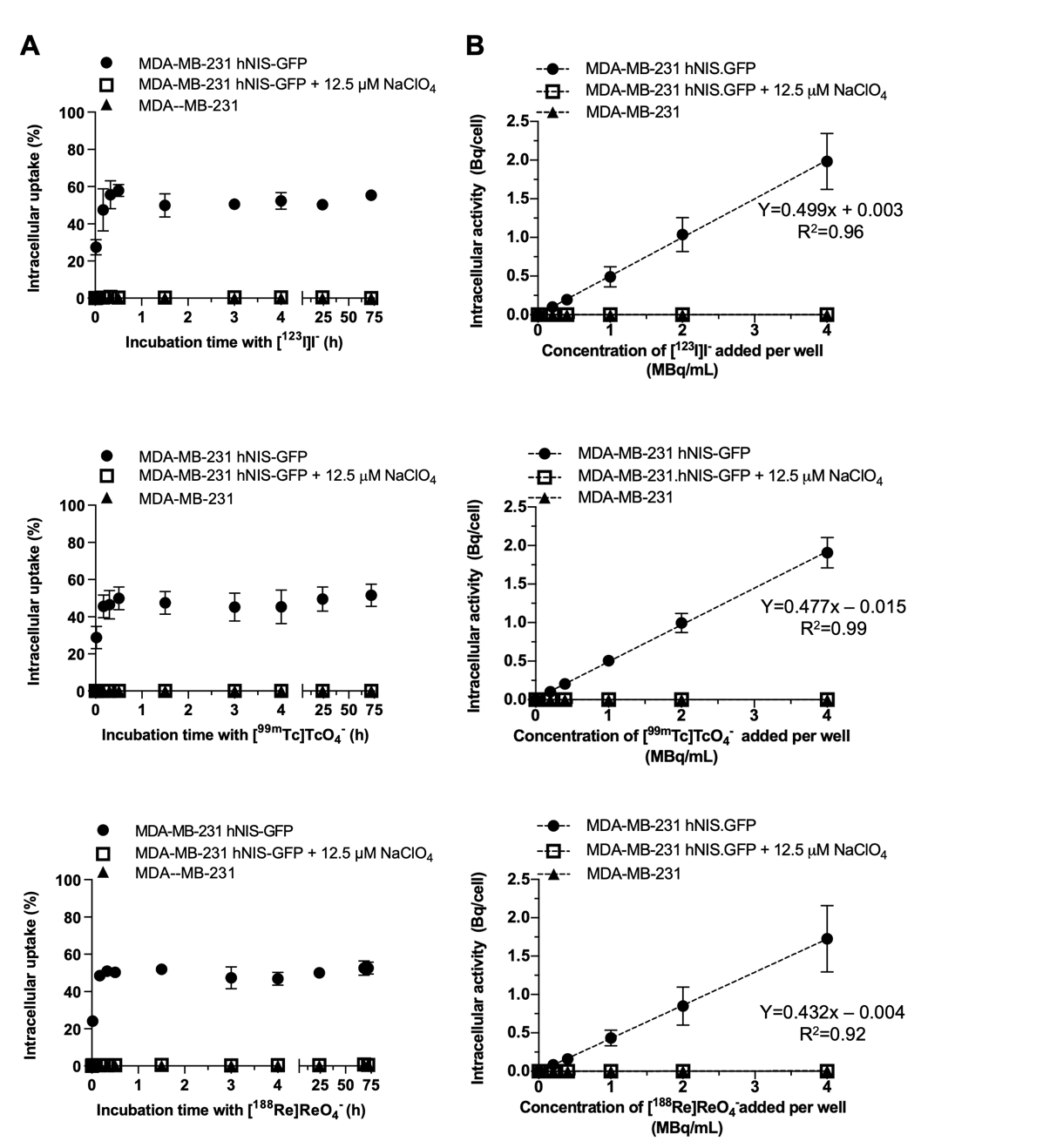
**

**Figure E4.** Intracellular uptake of [^123^I]I^─^, [^99m^Tc]TcO_4_^─^ and [^188^Re]ReO_4_^─^ is specific and mediated by hNIS. **(A)** Intracellular uptake percentage of radionuclides over time up to 72 hours with 0.2 MBq/mL into MDA-MB-231 and MDA-MB-231.hNIS-GFP cells in the presence or absence of NaClO_4_. **(B)** Intracellular radioactivity after 0.5-hour incubation with up to 4 MBq/mL of radionuclides in MDA-MB-231 and MDA-MB-231.hNIS-GFP cells in the presence and absence of NaClO_4_. Data is shown as average ± standard deviation (SD; N = 3-4 per group).

**
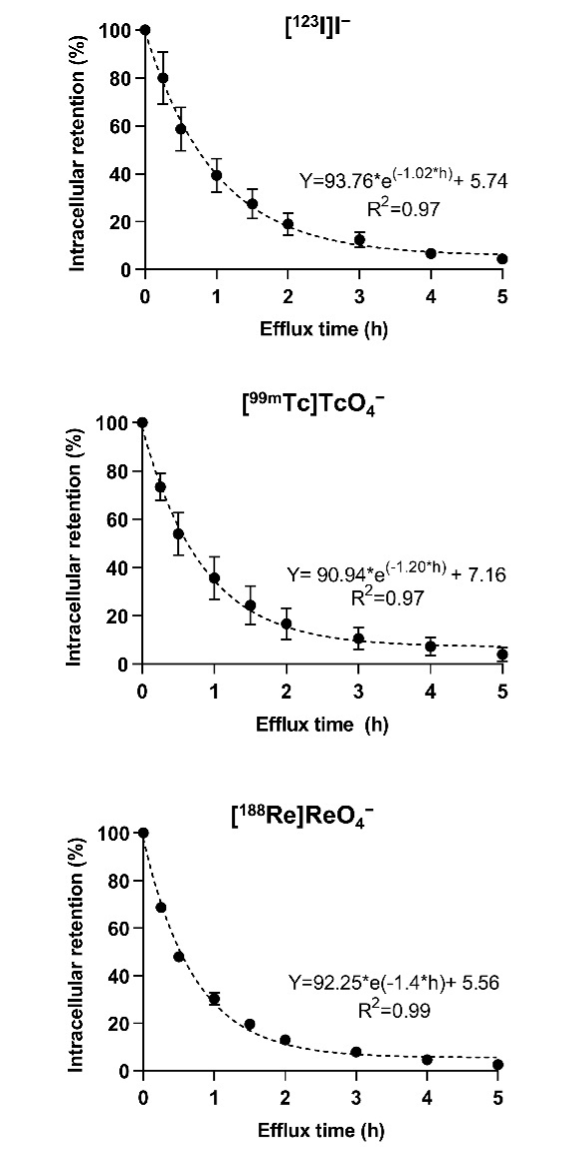
**

**Figure E5.** The intracellular retention of [^99m^Tc]TcO_4_^─^ [^123^I]I^─^ and [^188^Re]ReO_4_^─^ over time in MDA-MB-231.hNIS-GFP cells did not significantly differ. Graphs show intracellular retention percentage of radionuclides after an initial incubation of 0.2 MBq/mL for 0.5 hours. Data is shown as average ± SD, (N = 3-4/group).

**
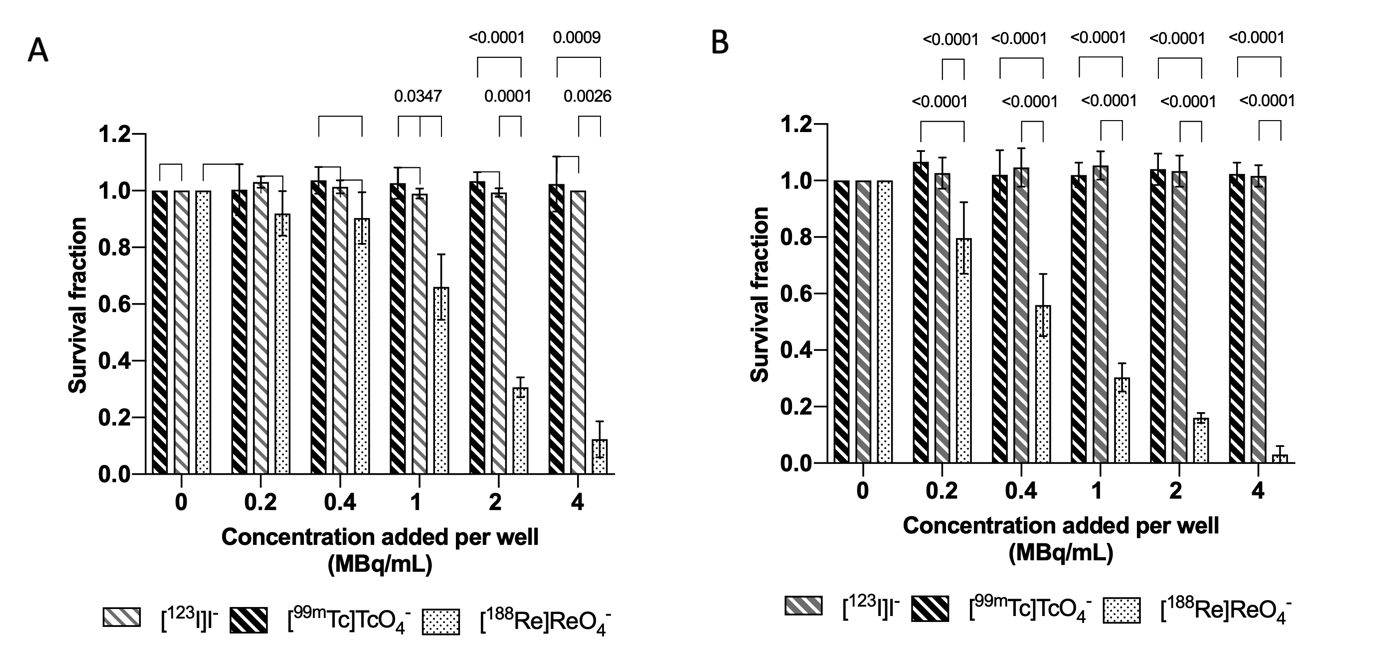
**

**Figure E6.** Radiotherapeutic effect induced by [^123^I]I^─^, [^99m^Tc]TcO_4_^─^ and [^188^Re]ReO_4_^─^ in the form of clonogenic survival. Survival fraction (SF) of MDA-MB-231 cells following **(A)** 24 hours and **(B)** 72 hours treatment with increasing activity concentrations of [^123^I]I**^─^**, [^99m^Tc]TcO_4_**^─^** and [^188^Re]ReO_4_**^─^** up to 4 MBq/mL. Data is shown as average ± SD, (N = 3-4/group).


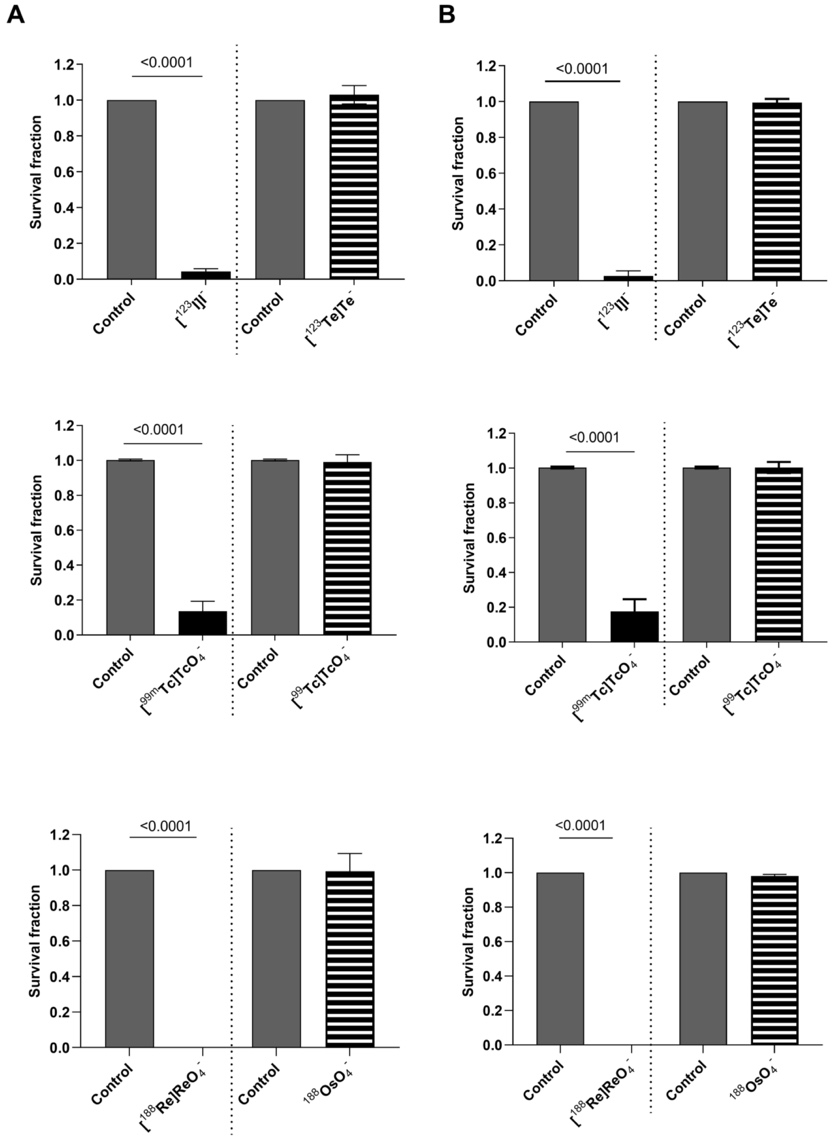


**Figure E7.** **Radiotherapeutic effect specificity of [^123^I]I^─^, [^99m^Tc]TcO_4_^─^ and [^1888^Re]ReO_4_^─^ in form of clonogenic survival.** Survival fraction of MDA-MB-231.hNIS-GFP following **(A)** 24 hours and **(B)** 72 hours with 4 MBq/mL of [^123^I]I**^─^,** [^99m^Tc]TcO_4_**^─^** and [^1888^Re]ReO_4_**^─^** and respective decayed daughter products [^123^Te]Te**^─^,** [^99^Tc]TcO_4_**^─^**, and ^188^OsO_4_**^─^** with the same concentration. Data is shown as average ± SD (*N* = 3).


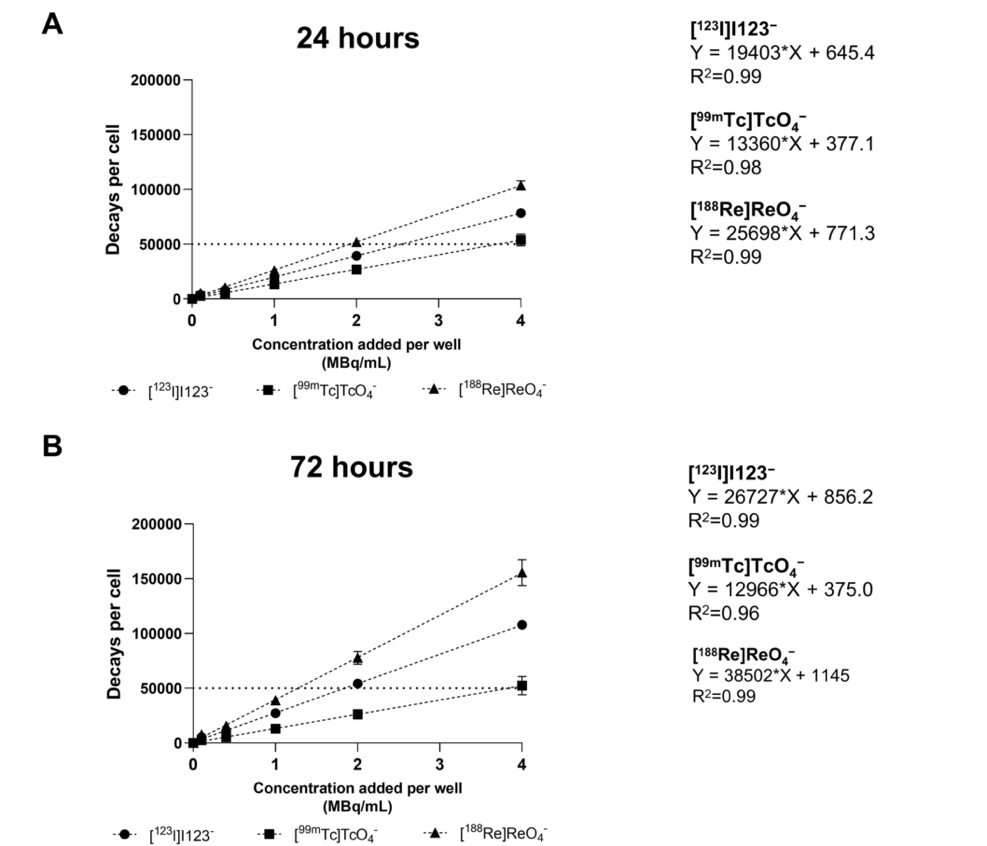


**Figure E8.** **Variation of the total number of decays per cell for the same radioactivity concentration added per well (MBq/well) and incubation time for each radionuclide.** Graphs illustrate the decays per cell in function of the concentration added per well (MBq/mL) following incubation with [^99m^Tc]TcO_4_^−^, [^123^I]I^−^ and [^188^Re]ReO_4_^−^ for **(A)** 24 and **(B)** 72 hours. Data was fitted with simple linear regression (dashed line). The dotted line helps visualize the concentration added per well at which 50,000 decays were obtained for each radionuclide. Data is shown as average ± SD (*N* = 3-4).


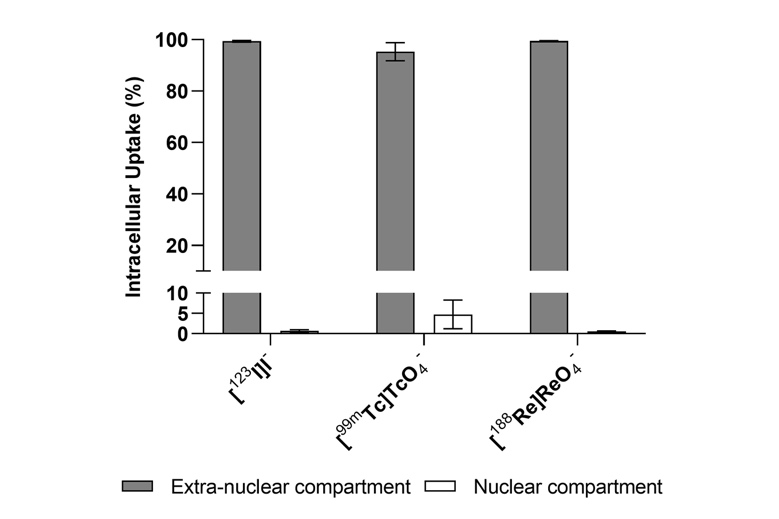


**Figure E9.** Cell fractionation method indicates that [^123^I]I^─^, [^99m^Tc]TcO_4_^─^, and [^188^Re]ReO_4_^─^  were localized in the extra-nuclear compartment**.** Data is shown as average ± SD (*N* = 3).


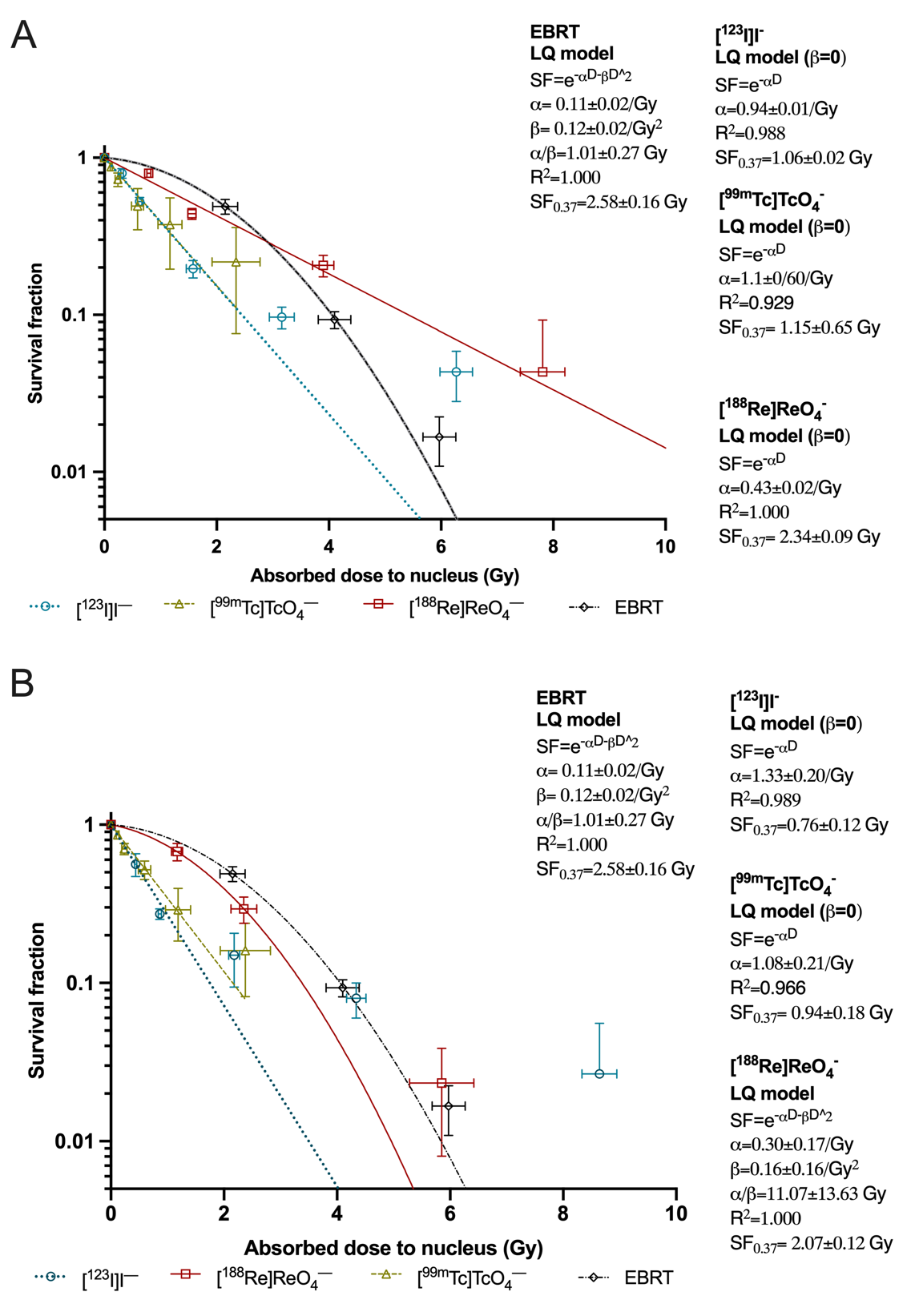


**Figure E10.** Dose-response curves of the survival fraction (SF) of MDA-MB-231.hNIS-GFP cells irradiated with EBRT up to 8 Gy or incubated with [^99m^Tc]TcO_4_^─^, [^123^I]I^─^ and [^188^Re]ReO_4_^─^ for (**E**) 24 hours and (**F**) 72 hours as a function of the estimated absorbed dose to nucleus (Gy) taking into account self- and cross-doses. N=3-4/group.


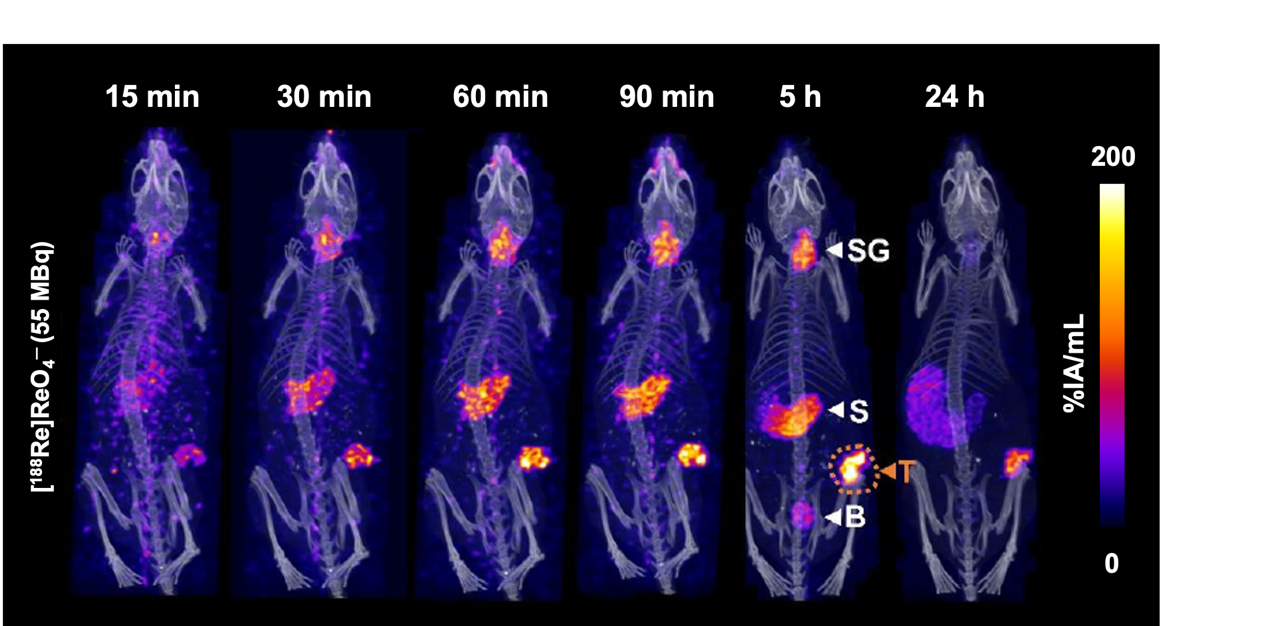


**Figure E11.** SPECT/CT image-based biodistribution of [^188^Re]ReO_4_^─^ up to 24 hours post intravenous administration of 55 MBq. Maximum intensity projections (MIPs) show the percentage activity per mL (% IA/mL) of radionuclides in endogenously NIS-expressing organs (white inscriptions: LG-lacrimal glands, Th- thyroid, and S-stomach), MDA-MB-231.hNIS-GFP tumors (orange inscription: T- tumor). CT images (grayscale) were used as anatomical reference.

**
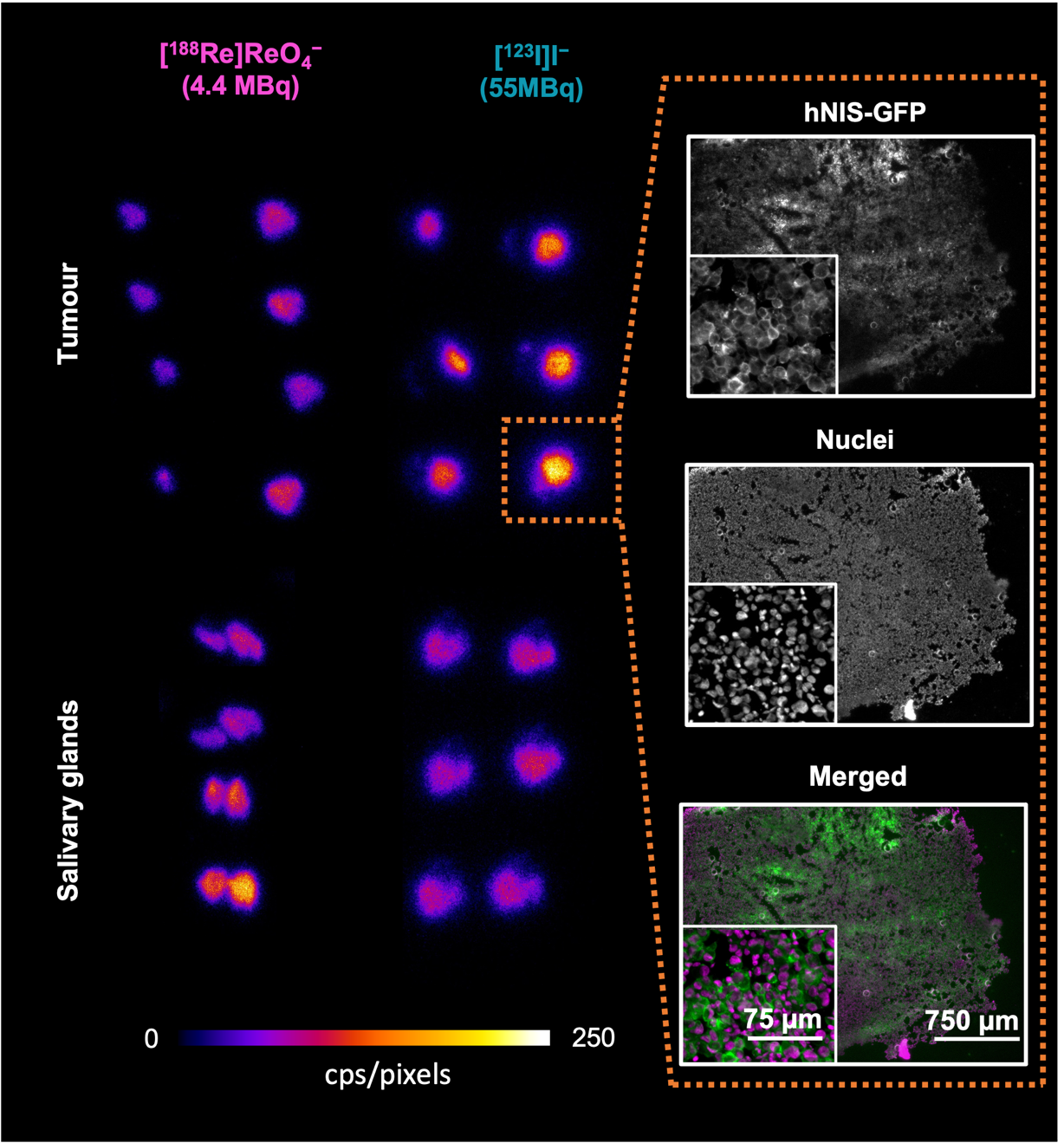
**

**Figure E12.** Immunofluorescence imaging shows predominant localisation of hNIS-GFP in the plasma membrane of MDA-MB-231.hNIS-GFP tumor sections and autoradiography shows that that radioactivity is not uniformly distributed in tissue sections. Immunofluorescence images of 10 µm thickness sections of MDA-MB-231.hNIS-GFP tumor xenografts show hNIS-GFP depicted by green colour and nuclei (DAPI staining) by purple colour. CPS = counts per second. *N* = 4.

**
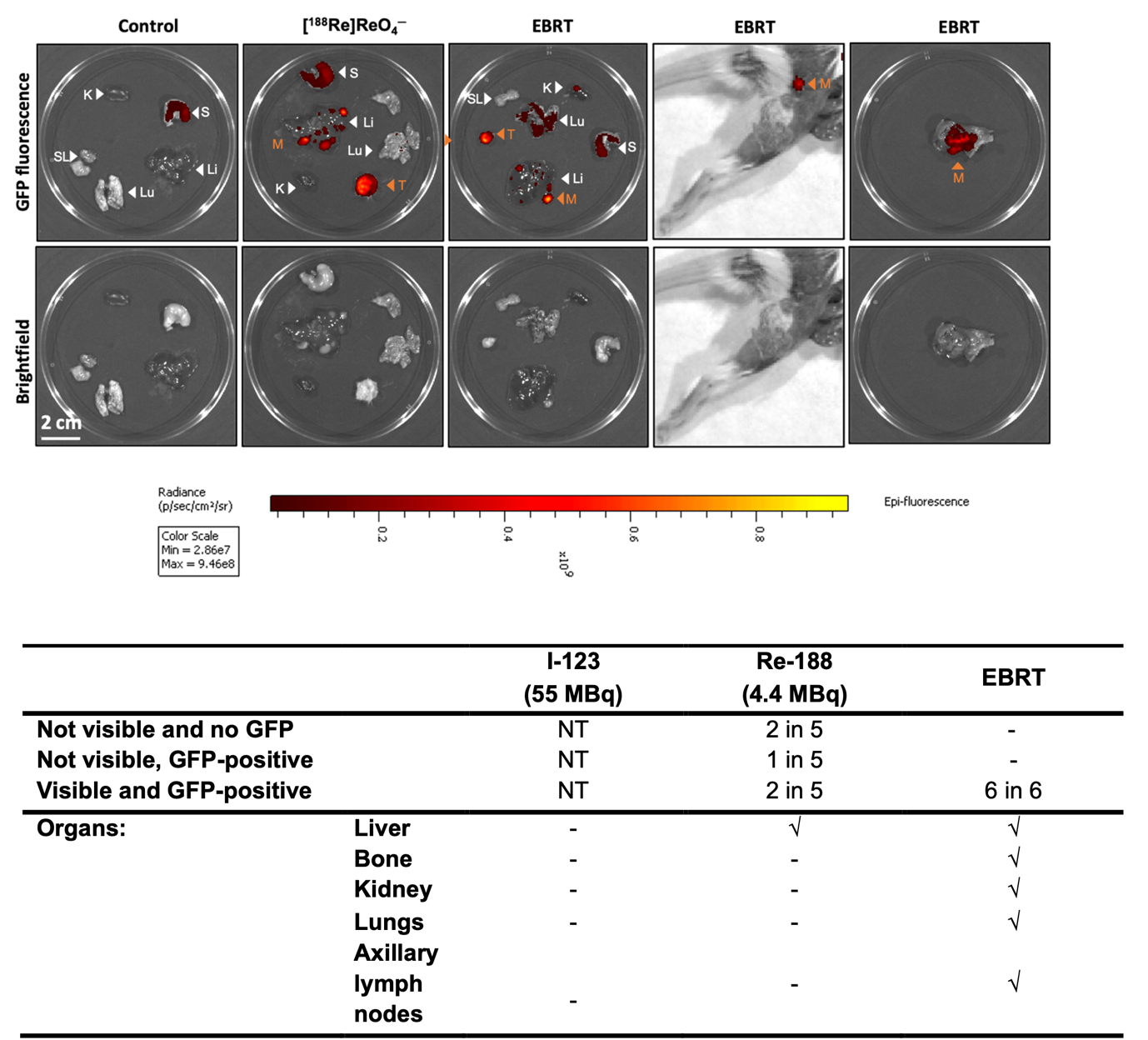
**

**Figure E13.** Representative GFP fluorescence imaging of hNIS-expressing MDA-MB-231 tumors and healthy organs reveal the development of MDA-MB-231.hNIS-GFP metastases (orange inscriptions: T-tumor and M-metastases) and healthy organs (white inscriptions: K- kidney, S-stomach, SL- salivary glands, Li- Liver, and L-lungs) at the time that studies were terminated. Tissues were imaged for GFP fluorescence using IVIS^®^ Spectrum (PerkinElmer^®^, USA). Table shows observations of GFP-fluorescent metastases in tissues collected for mice irradiated with EBRT (N=6) and administered with 55 MBq [^123^I]I^─^ or 4.4 MBq [^188^Re]ReO_4_^─^ (N=5 per radionuclide). NT = not tested.

**References**

1. Blower PJ. Extending the life of a 99Tcm generator: a simple and convenient method for concentrating generator eluate for clinical use. Nucl Med Commun. 1993;14(11):995-7.

2. Vaziri B, Wu H, Dhawan AP, Du P, Howell RW, Committee SM. MIRD pamphlet No. 25: MIRDcell V2.0 software tool for dosimetric analysis of biologic response of multicellular populations. J Nucl Med. 2014;55(9):1557-64.

3. TruongVo TN, Kennedy RM, Chen H, Chen A, Berndt A, Agarwal M, et al. Microfluidic channel for characterizing normal and breast cancer cells. Journal of Micromechanics and Microengineering. 2017;27.

4. Basic anatomical and physiological data for use in radiological protection: reference values. A report of age- and gender-related differences in the anatomical and physiological characteristics of reference individuals. ICRP Publication 89. Ann ICRP. 2002;32(3-4):5-265.
